# Photoluminescence lifetime stability studies of β‐diketonate europium complexes based phenanthroline derivatives in poly(methyl methacrylate) films

**DOI:** 10.1002/open.202300192

**Published:** 2024-01-12

**Authors:** Othmane Essahili, Mouad Ouafi, Mohamed Ilsouk, Omar Lakbita, Carine Duhayon, Lhassane Mahi, Omar Moudam

**Affiliations:** ^1^ Applied Chemistry and Engineering Research Centre of Excellence (ACER CoE) Mohammed VI Polytechnic University (UM6P) Lot 660, Hay Moulay Rachid 43150 Benguerir Morocco; ^2^ Laboratoire de Chimie de Coordination du CNRS UPR 8241 205, route de Narbonne, BP 44099 F-31077 Toulouse Cedex 4 France; ^3^ MAScIR CCI Mohammed VI Polytechnic University (UM6P) Lot 660, Hay Moulay Rachid 43150 Benguerir Morocco

**Keywords:** β-diketonate europium complexes, Long-term stability, Phenanthroline derivatives ligands, Photoluminescence stability, PMMA films

## Abstract

In this work, five phenanthroline derivatives substituted with different methyl groups have been selected to synthesize β‐diketonate‐based europium complexes to check the influence of the substitutions on the degradation effect of those complexes in poly(methyl methacrylate) (PMMA) films. The photophysical properties of Eu(III) complexes, including absorbance, excitation, and emission have been carefully investigated in solution, solid‐state, and doped in PMMA film. In all these states, the complexes exhibit an impressive red emission at 614 nm with a high photoluminescence quantum yield of up to 85 %. The films have been exposed under outdoor, indoor, and dark storage stability lifetime conditions for 1200 hours. The photoluminescence measurements recorded every 400, 800, and 1200 hours demonstrated that the film containing europium complex with phenanthroline ligand substituted by a high number of methyl groups (Eu(TTA)_3_L_5_) showed good photoluminescent stability in indoor and dark conditions, and exhibited better resistance to degradation in outdoor conditions compared to other complexes. This study has proved that phenanthroline ligands could be tuned chemically leading to better stability of those types of complexes in films which can be end‐used for future stable optoelectronic devices such as luminescent solar concentrators.

## Graphical abstract

This study contributes to a deeper understanding of the design and development of stable β‐diketonate europium complexes based on phenanthroline ligands in PMMA films. The synthesis, characterization, thermal, and photoluminescence properties are provided accompanied by long lifetime stability tests in different conditions which indicate the potential for these complexes as candidates for future optoelectronic devices, including luminescent solar concentrators.

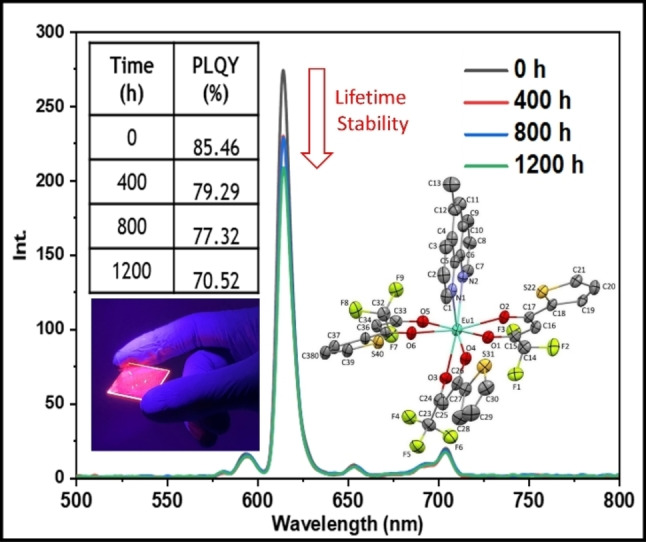

## Introduction

In recent years, a great deal of attention has been focused on lanthanide (Ln) luminescent complexes due to their varied applications in luminescent solar concentrators,[^1^, ^2^] sensors, OLEDs, lasers, and display devices.[^3^, ^4^, ^5^, ^6^] These materials feature distinct characteristics such as narrow emission peaks, large Stokes shift, and longer lifetimes of the emissive state.[^7^, ^8^] However, the direct excitation of trivalent lanthanide ions by *4f–4f* transitions is difficult due to their low molar absorption coefficient and parity‐gap nature.^[9]^ To improve emission intensity, organic chromophores with high absorption coefficients are used to coordinate lanthanide ions, facilitating efficient energy transfer via an “antenna effect”. These chromophores also protect the emission from non‐radiative deactivation. Choosing an effective sensitizer for lanthanide ions is a complex task, and β‐diketonates have proved to be a suitable class of chromophores.^[10]^

β‐diketonate based lanthanide complexes have been widely studied due to intense luminescence. However, these complexes are often coordinatively unsaturated and solvated, resulting in non‐radiative energy loss.^[11]^ Coordinative saturation can be achieved by incorporating neutral auxiliary ligands containing oxygen/nitrogen donor atoms, leading to coordinatively saturated lanthanide β‐diketonate complexes. These complexes exhibit high quantum yields and are suitable for luminescent devices. Europium complexes, known for their red emission, have found applications in display devices, luminescent solar concentrators, and sensors. Incorporating organic europium structures into suitable substrates produces excellent luminescent materials capable of emitting white light by varying the excitation wavelength.[^12^, ^13^] To ensure the manufacture of stable, long‐lasting optoelectronic devices, the photostability of luminescent lanthanide complexes can be enhanced by incorporating them into polymer matrices such as polymethyl methacrylate (PMMA).^[14]^

This study focuses on conducting a comprehensive investigation into the long‐term stability of europium β‐diketonate complexes. These complexes utilize 2‐Thenoyltrifluoroacetone (TTA) as the primary ligand and employ phenanthroline derivatives as auxiliary ligands. This research aims to explore the impact of phenanthroline modification with methyl (−CH_3_) groups on the photoluminescence stability of PMMA‐doped films incorporating these complexes over an extended period. The primary objective is to evaluate the photoluminescence stability of five PMMA‐doped complexes under diverse conditions, including darkness, indoor and outdoor environments, humidity, and direct light exposure.

## Results and Discussion

### Synthesis of Eu(TTA)_3_L_1‐5_ complexes

**Scheme 1:**
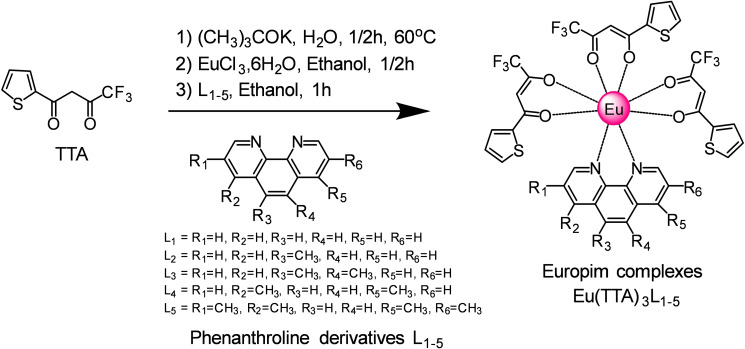
Synthesis of β‐diketonate europium complexes.

**Figure 1:**
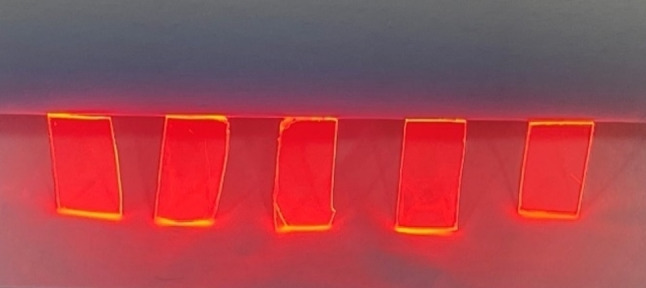
Strong visible red emission of Eu‐doped films in PMMA under UV light.

Eu(TTA)_3_L_1‐5_ complexes have been synthesized from three equivalent of 2‐Thenoyltrifluoroacetone (TTA) ligands in a mixture with one equivalent of phenanthroline derivative ligand (L_1‐5_) in the presence of europium (III) chloride hexahydrate as shown in Scheme 1. The TTA is mixed first with potassium tert‐butoxide (tBuOK) in water at 60 °C under nitrogen, followed by the addition of europium (III) chloride hexahydrate, the correspondent phenanthroline derivative ligand is added in ethanol to form the β‐diketonate europium complexes Eu(TTA)_3_L_1‐5_ as explained in Scheme 1.^[7]^ The europium ion is coordinated with six oxygen atoms from three TTA ligands and two nitrogen atoms from the phenanthroline ligand, the structures are already confirmed from the literature and demonstrated here in this work with the help of the single XRD in the next section. Furthermore, when any europium complex is embedded in polymethylmethacrylate, it forms a very transparent film with very high red emission when exposed to UV light as shown in Figure 1. The embedded europium complex in PMMA film absorbs the irradiated UV light and re‐emits a red visible light by luminescent down‐shifting concept, the red light diffused throughout the film and trapped to the edges by attuned total reflection leading to a clear visible concentration of the light at the edges of the films.

### Single XRD structure of the complexes

**Figure 2:**
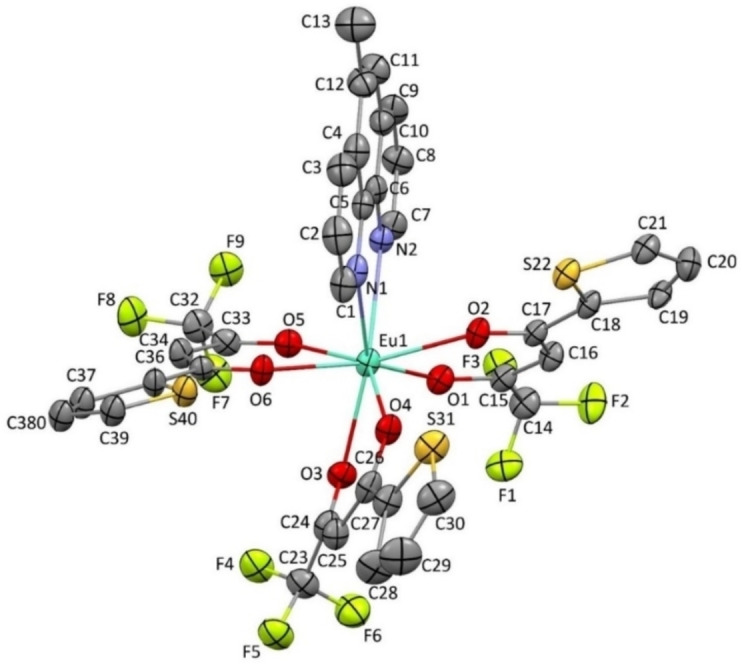
Mercury projection of [Eu(TTA)_3_L_2_] complex. Hydrogen atoms are deleted for clarity.

**Figure 3:**
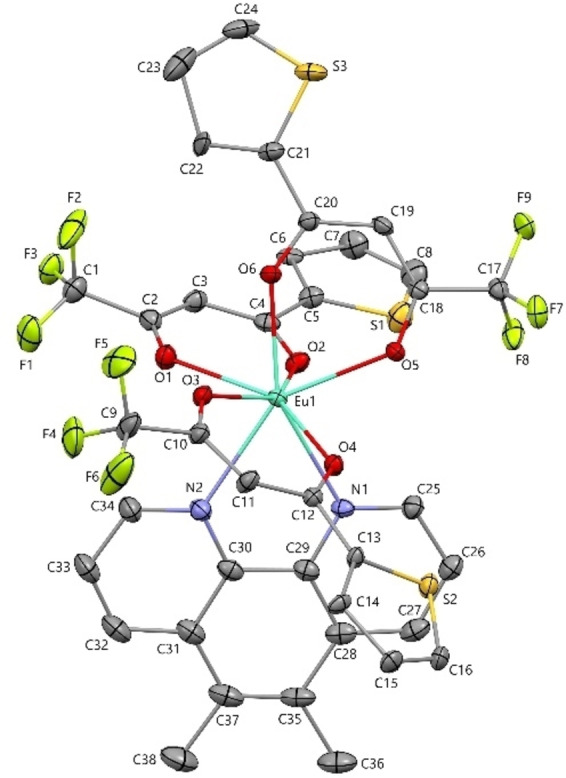
Mercury projection of [Eu(TTA)_3_L_3_] complex. Hydrogen atoms are deleted for clarity.

The Mercury projection of the two europium complexes Eu(TTA)_3_L_2_ and Eu(TTA)_3_L_3_ in Figures 2 and 3 illustrated that the complexes possesses a coordination sphere geometry of eight bonds, six Eu−O bonds with the 2‐Thenoyltrifluoroacetone (TTA) ligands, and two Eu−N bonds with the phenanthroline derivative ligands. For the Eu(TTA)_3_L_2_ complex, the average distances of Eu−O and Eu−N bonds correspond to 2.36 Å and 2.59 Å, respectively,^[15]^ while the coordination bite angles of N−Eu‐N and O−Eu‐O are in the ranges of 62.67° and 72.15° (mean value), respectively.^[15]^ For Eu(TTA)_3_L_3_ complex, the bonds distances and angles are in the same ranges. To delve deeper into the macromolecular interaction in the crystal structure of the complex, Mercury projection of Eu(TTA)_3_L_2_ demonstrated that a π–π stacking interaction is noticeable between two aromatic rings of the superposed and adjacent phenanthrolines ligands of the europium complex, as illustrated in Figure S1. Due to this famous interaction,^[16]^ the phenanthroline ligand is slightly distorted. Furthermore, Figure S2 shows that two TTA ligands counterpose due to equal reverse interactions between CF_3_ and thiophenes from the two different TTA adjacent ligands.

### NMR Spectra of the complexes Eu(TTA)_3_ L_1‐5_

**Figure 4:**
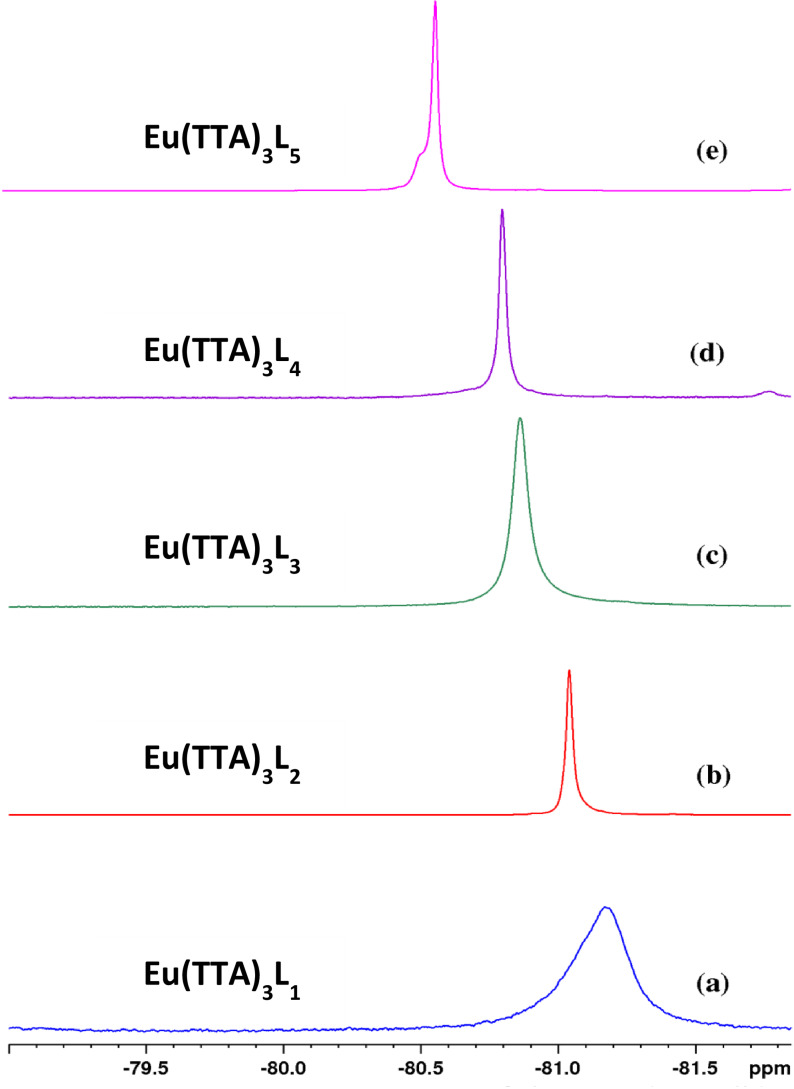
^13^F‐NMR of Eu(TTA)_3_L_1‐5_ complexes.

^19^F‐NMR spectra of the europium complexes were carried out in CDCl_3_ solutions at room temperature. All complexes show a signal (Figure 4) assigned to the fluorine atom of the TTA ligand with a multiplicity value of 9 due to the presence of three TTA molecules in each complex. The chemical shift of Eu(TTA)_3_ L_1‐5_ complexes are −81.18, −81.04, −80.86, −80.79, and −80.56 ppm, respectively. When we switch from the Eu(TTA)_3_L_1_ complex to the Eu(TTA)_3_L_5_ complex, the number of CH_3_ donor groups increases, which results in a chemical shift towards the larger magnetic fields, and normally when the CH_3_ donor groups are added in the organic molecules, the chemical shift moves towards the lower magnetic field, and it is the opposite in this case. This may be due to the paramagnetic effect of the europium complex on the magnetic field.

The successive substitutions of the protons of phenanthroline by methyl groups affect in particular the paramagnetic deshielding effects of the europium atom and modify the polarization in the vicinity of the fluorine atoms. The NMR chemical shift of the latter is thus increasingly higher (Figure 4 spectra (a)–(e)).

The deshielding paramagnetic contribution is linked to the type of Metal−Ligand bonding orbitals and is modulated by relativistic spin‐orbit effects.[^17^, ^18^, ^19^, ^20^, ^21^] Any change of the chemical environment of one of the ligands (change in nature of neighboring atoms, repositioning them, or substituting them with others),[^22^, ^23^, ^24^, ^25^] on either side of the metal atom, induces a significant repolarization of the same orbital on the side of the second ligand.[^26^, ^27^] Both atom ligands thus mutually influence their bonding character with the metal,^[28]^ and in turn influence the ligand NMR deshielding of the other neighboring atoms of the complex.

Thus, in the present work, the nitrogen‐oxygen atoms for the Eu‐complexes (L_2_‐L_5_), and their respective neighboring atoms, influence the NMR paramagnetic deshielding mechanisms via the Eu−O and Eu−N bonds. Eu's deshielding effect is enhanced by the electronegativity of the two oxygen atoms, hence the significant shifts observed on the fluorine NMR spectra of complexes studied, Figure 4.

Moreover, this paramagnetic deshielding is more accentuated as the character of the Eu−N bond is affected by the substitution of the hydrogen aromatic rings of the phenanthroline by methyl groups. The electropositive effect of the latter is transmitted through the aromatic rings and leads to an alteration of the Eu−N bonds polarization. This alteration is then transferred to the oxygen atoms via the orbitals shared by the europium atom and the two light atoms (N−Eu−O). The deshielding of the fluorine atoms is therefore further enhanced and the chemical shifts of fluorine in the NMR spectra of the complexes are increasingly high.

### FTIR spectra of the complexes Eu(TTA)_3_ L_1‐5_

**Figure 5:**
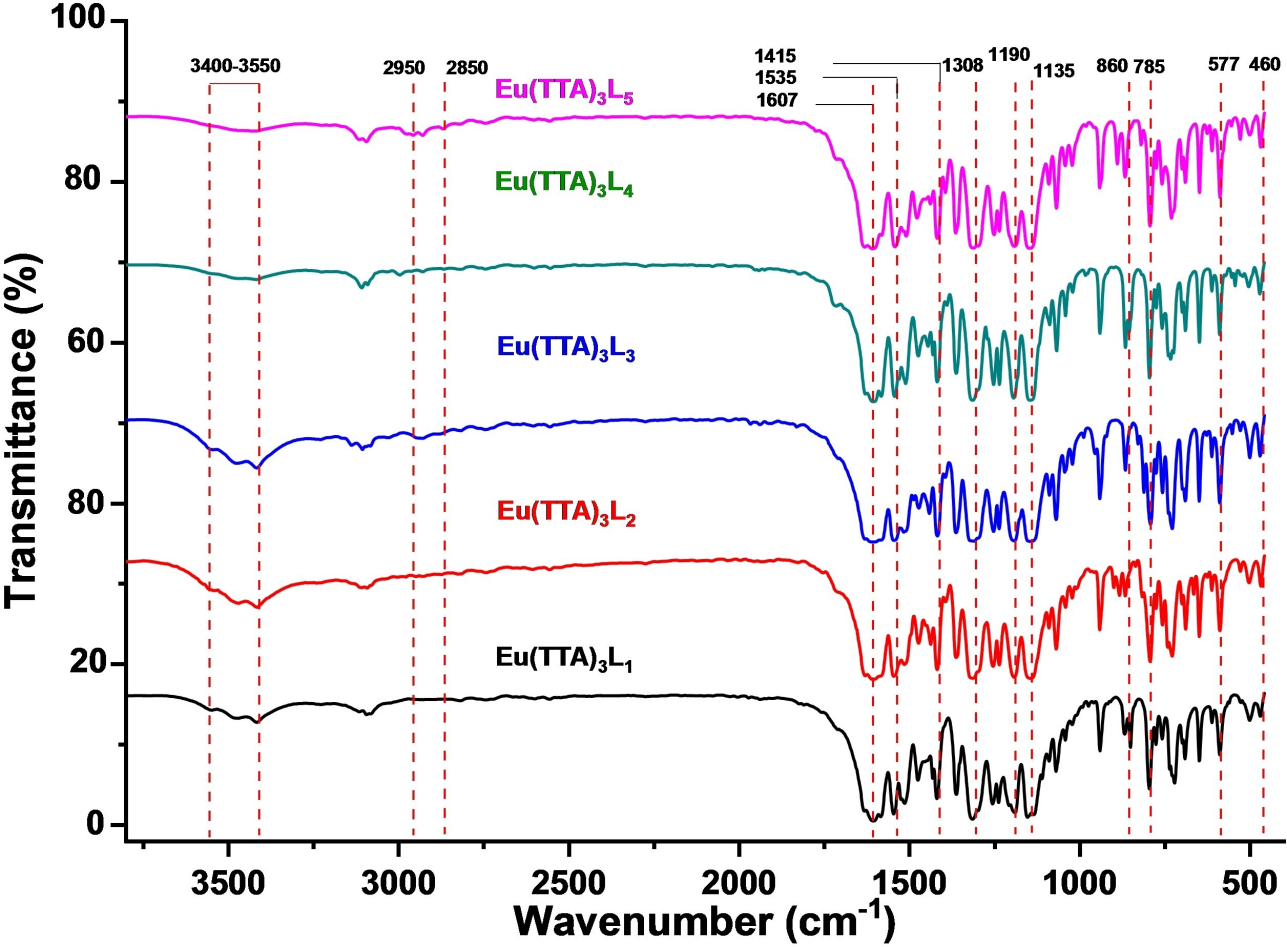
FTIR spectra of the complexes Eu(TTA)_3_ L_1‐5_

The FTIR spectra of europium complexes (Eu (TTA)_3_ L_1‐5_) are shown in the Figure 5. Since the spectra of all complexes are similar, Eu (TTA)_3_L_5_ is selected to discuss the different absorption bands. The spectrum displays characteristic bands at 2950 and 2850 cm^−1^, corresponding to asymmetric and symmetric valence vibrations of aliphatic C−H stretching (−CH₃) groups. These peaks reveal the presence of methyl groups in the chemical structure of phenanthroline ligand. The FTIR spectrum shows also an intense peak emerges around 1630 cm^−1^, attributed to the carbonyl group (C=O) stretching vibration, while another peak at 1535 cm^−1^ corresponds to (C=C) stretching. These bands serve as indicative signals of the presence of carbonyl groups and double bonds of the TTA ligand. The absorption peak at 1415 cm^−1^ is associated with the stretching vibration of the (C=N) bond of the ligand coordinated to the Eu ion.^[29]^ This peak is significant in confirming the coordination of the ligands with the europium ion. New frequency bands emerge around 577 and 460 cm^−1^, providing evidence for the existence of Eu−N and Eu−O bonds, respectively.^[30]^ These findings strongly support the coordination of the ligands with the europium ion, emphasizing the formation of specific metal‐ligand interactions. Further exploration reveals additional peaks at approximately 1308 and 1190 cm^−1^ indicating the presence of (C−F) and (C−CF₃) groups of the ligand (TTA). These bands contribute to the characterization of the ligand structure in the complexes.^[31]^ Bands at 785 and 860 cm^−1^ are assigned to the aromatic rings present in the complexes. Additionally, a broad characteristic band at around 3400–3550 cm^−1^ is observed, indicating the presence of the hydroxyl group (−OH). This band is attributed to the presence of water or ethanol molecules, which probably originate from the sample preparation process.

### Thermal stability

**Figure 6:**
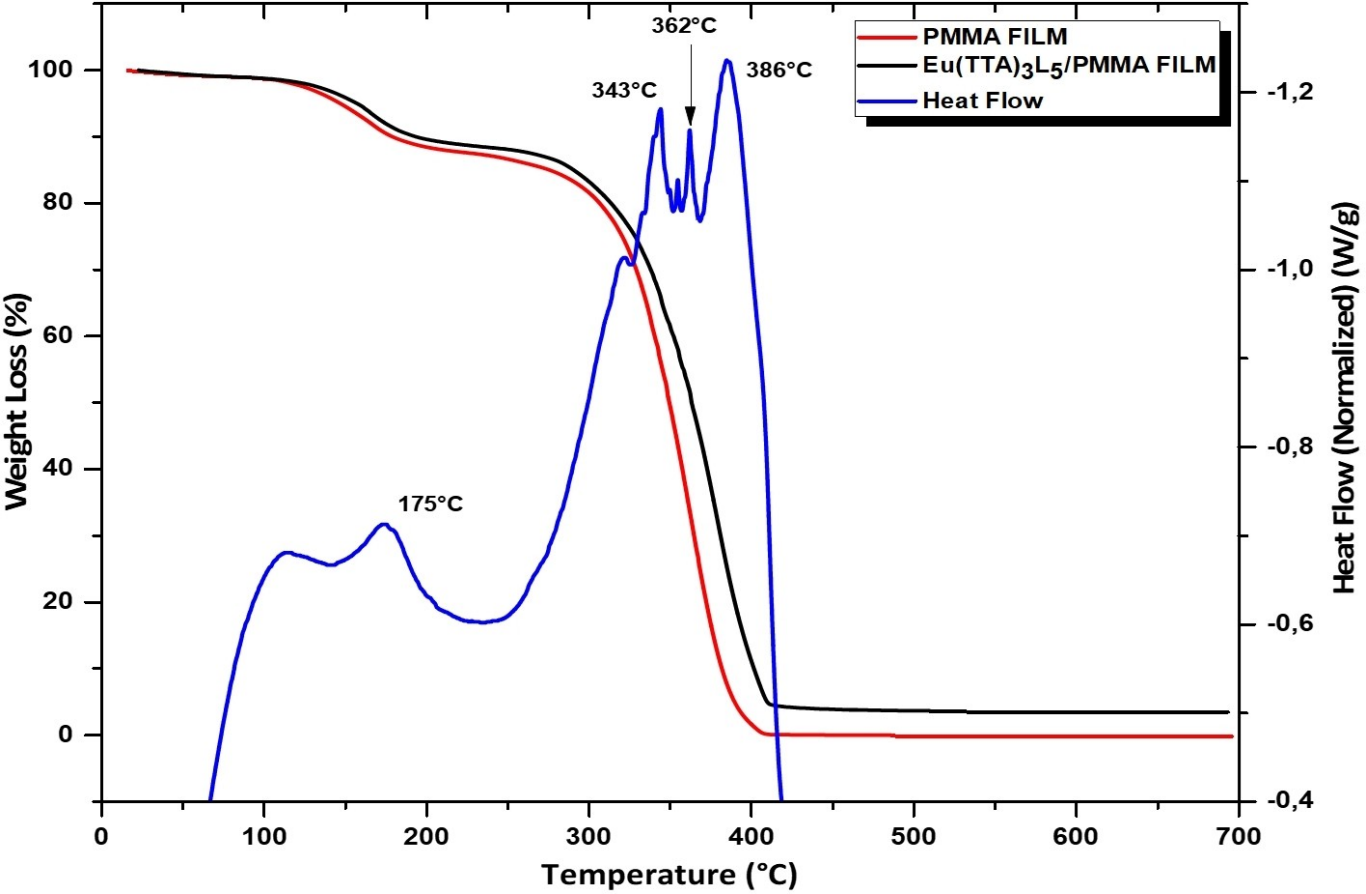
Thermogravimetric (TGA) and differential thermogravimetric (DTA) curves of PMMA film and its respective Eu(TTA)_3_L_5_/PMMA film.

Thermogravimetric analysis (TGA) was used to study the degradation behavior of Eu(TTA)_3_L_1‐5_ complexes and the corresponding Eu(TTA)_3_L_1‐5_/(PMMA) films, as illustrated in Figure 6. The thermal behavior of PMMA films incorporating all five europium complexes showed notable similarities in their TGA curves. To illustrate the consistent thermal behavior observed for all complexes in the same series, TGA‐DTA thermograms of pure PMMA and a PMMA film incorporating the Eu(TTA) _3_L_5_ complex were chosen for demonstration. Both thermograms show two distinct decomposition stages. The initial weight loss, occurring below 175 °C, was attributed to the release of physically adsorbed water and residual solvents, estimated at around 10 %.^[32]^ The later weight loss, in the temperature range from 250 to 450 °C, was associated with the dissociation of organic ligand molecules (TTA and phenanthroline derivatives) from the complexes at around 342 and 362 °C.^[31]^ Furthermore, the largest exothermic peak observed at 386 °C corresponds to the thermal decomposition behavior of PMMA.

These results confirm that the thermal stability of the PMMA film is considerably enhanced by complex doping, providing excellent thermal stability to advances in the research and development of optoelectronic devices.

### Optical properties and photoluminescence data of the complexes

**Figure 7:**
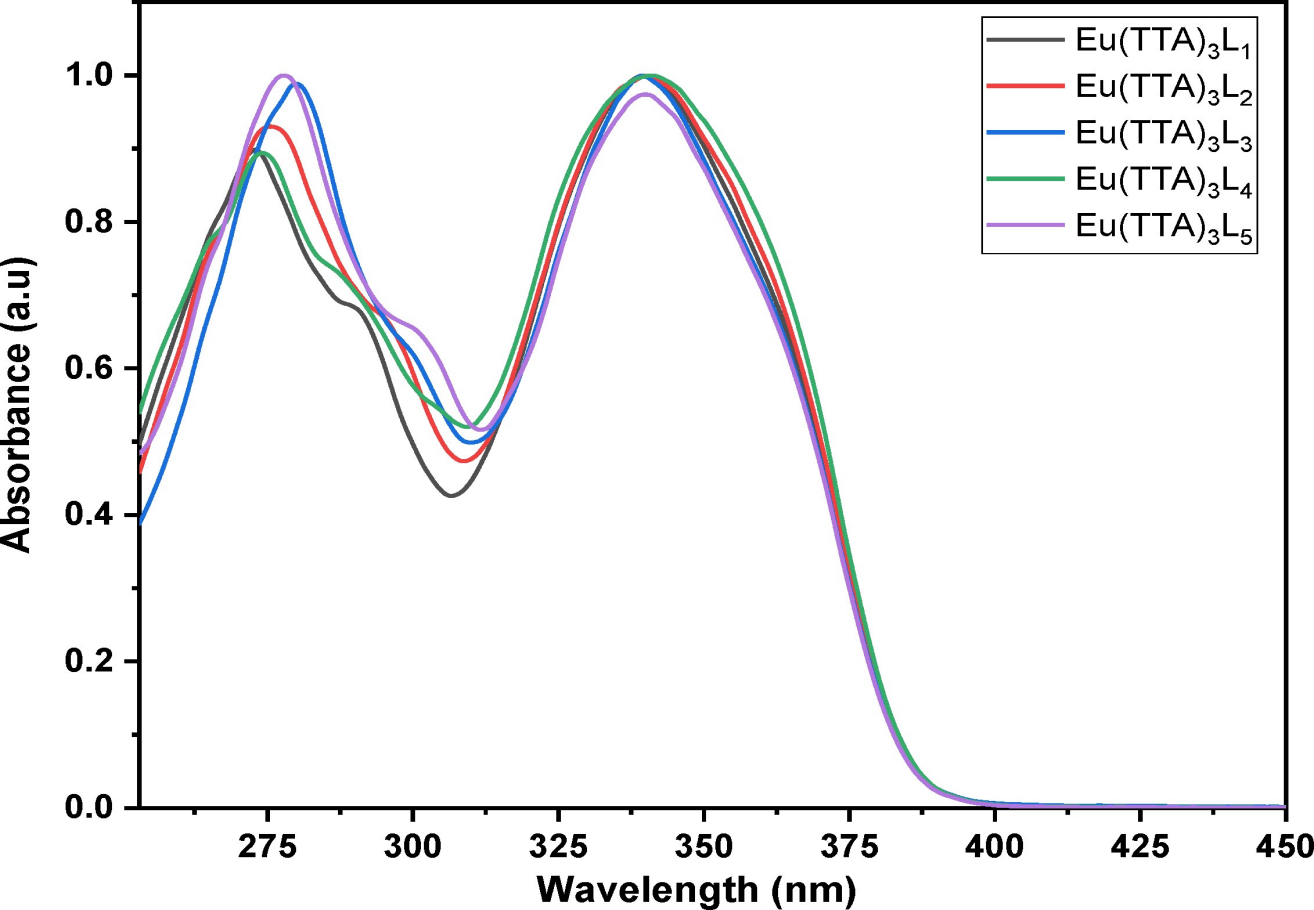
Normalized UV‐Vis absorption of Eu(TTA)_3_L_1‐5_ complexes in DCM solution.

The UV/Vis absorption spectra of the five complexes Eu(TTA)_3_ L_1‐5_ in DCM solution with a concentration value of 1.0×10^−5^ mol L^−1^ is shown in Figure 7. The absorption spectrum of complexes shows absorption from λ=250 to 400 nm with λ_max_ values of 279 and 348 nm, and these bands are attributed to the π→π* transitions.

**Figure 8:**
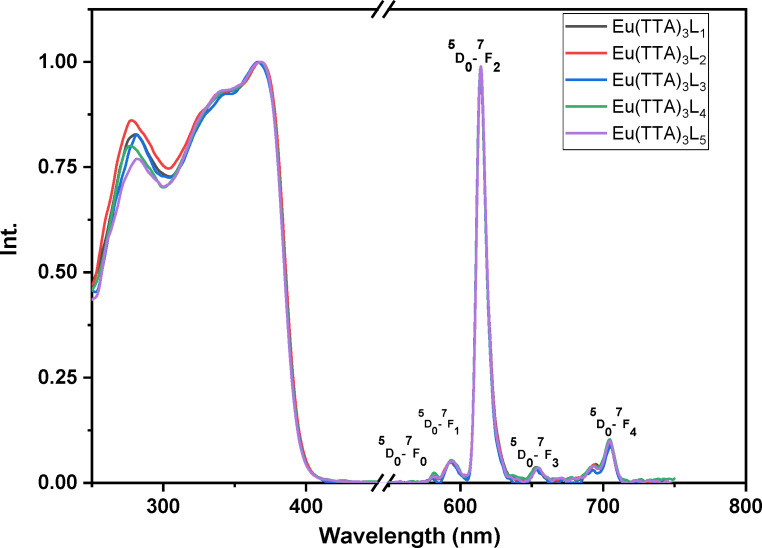
Normalized excitation and emission spectra of the complexes in DCM solution.

**Figure 9:**
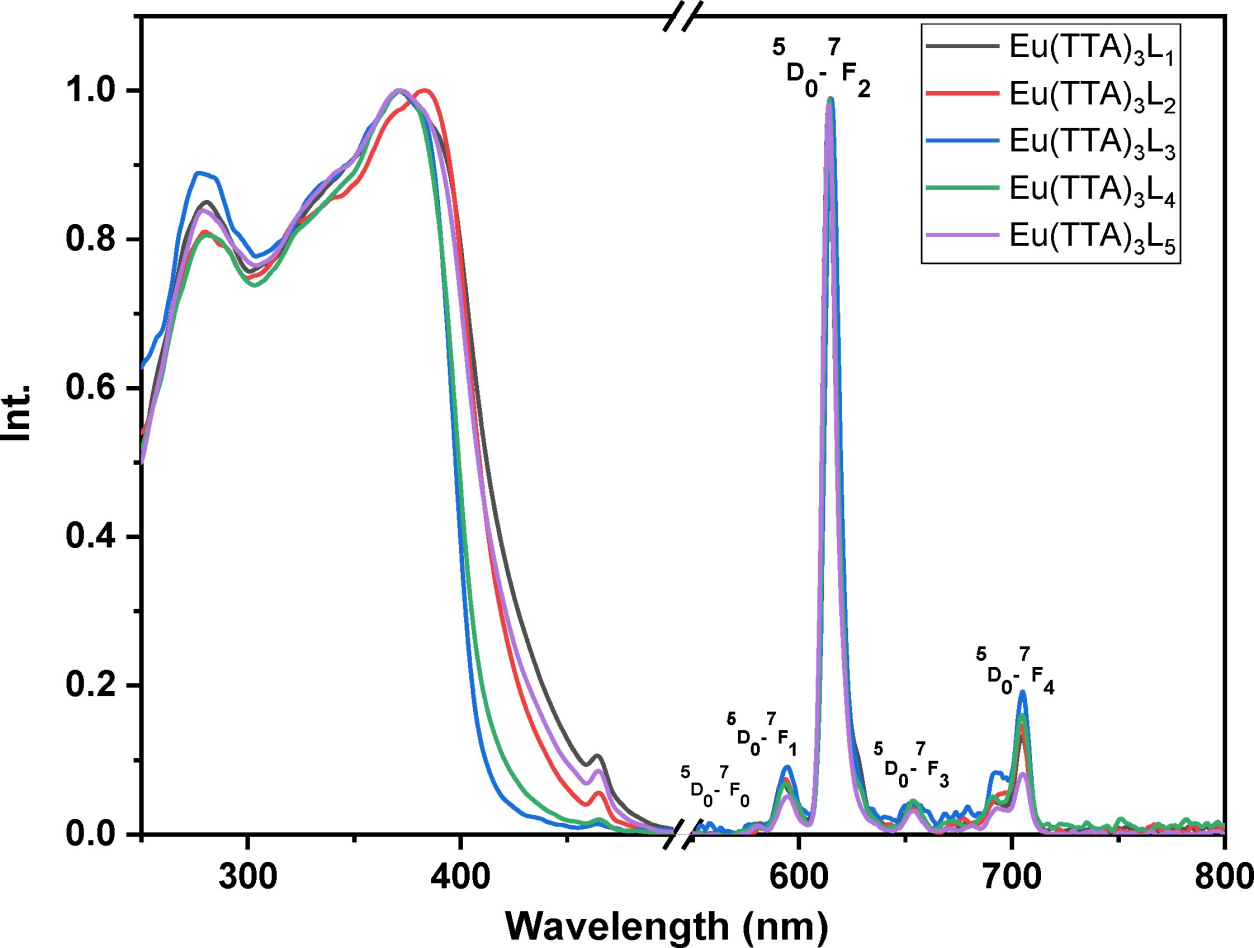
Normalized excitation and emission spectra of the complexes in solid‐state.

**Figure 10:**
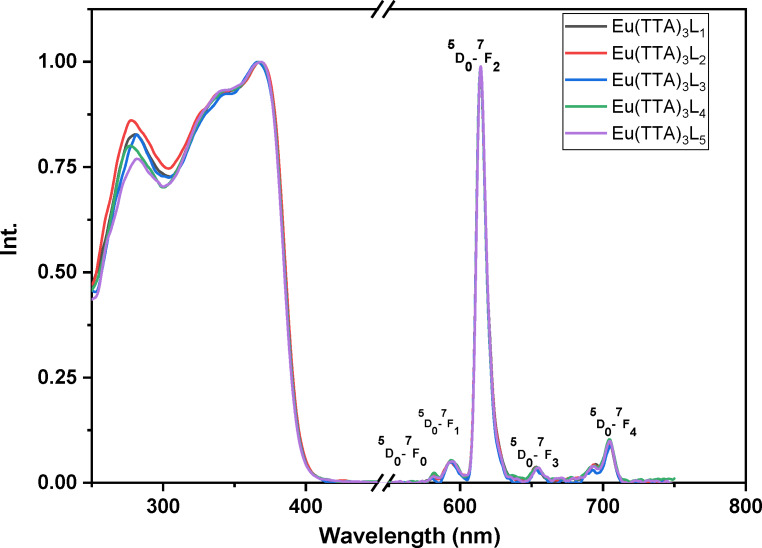
Normalized excitation and emission spectra of the complexes in PMMA matrix.

The excitation and emission spectra of the complexes Eu (TTA)_3_L_1‐5_ in solution (dichloromethane solution conc. 4.7×10^−4^ mol.l^−1^), in the solid phase and in the PMMA matrix are shown in Figures 8, 9, 10, respectively. All the complexes in the three different states show a broad excitation from 250 nm to 400 nm in solution, from 250 nm to 450 nm in the PMMA matrix, and from 250 nm to 480 nm in the solid‐state with maximum excitation wavelengths of 371, 378 and 382 nm respectively. This excitation is due to the π‐π* transitions of the TTA and Phenanthroline derivatives ligands.^[33]^ The bathochromic excitation shift observed in the solid‐state compared to those in solution and in the PMMA matrix is due to the aggregation of the complexes in the solid phase.^[34]^ After excitation of the ligand, the excitation energy is transferred to the lowest excited state ^5^D_0_ level of the Eu(III) ion, where an emission of different colors occurs. While the intensity of the red emission (electric or magnetic dipolar transition) depends on the local symmetry of the Eu ^III^ ion site in the complexes. As shown in Figures 8, 9, and 10, the complexes Eu (TTA)_3_L_1‐5_ exhibit emission bands at 580 nm, 591 nm, 614 nm, 651 nm, and 700 nm respectively correspond to ^5^D_0_→^7^F_0_, ^5^D_0_→^7^F_1_, ^5^D_0_→^7^F_2_, ^5^D_0_→^7^F_3_ and ^5^D_0_→^7^F_4_ transitions of Eu^3+^ ion. The emission peak at λ ≈612 nm for all complexes is an electric dipole transition and it is the hypersensitive transition, which is translated to intense red light, also indicating that the energy transfer from ligand Eu^3+^ is very effective.[^35^, ^36^] The weak emissions at 581 nm, 591 nm, 652, and 702 nm correspond to ^5^D_0_→^7^F_0_, ^5^D_0_→^7^F_1_, ^5^D_0_→^7^F_3_, and ^5^D_0_→^7^F_4_ transitions, respectively,^,[36][37]^ are magnetic transitions and, therefore, are not sensitive to the Eu^3+^ environment but, they are weak because they are strictly forbidden both in magnetic and electric dipole transitions according to the Laporte rule. It is clear from the excitation and emission spectra that the complexes exhibit a significant Stokes shift around 200 nm, as evidenced by the no overlap between the excitation and emission spectra, meaning that the self‐absorption loss is zero.

The Photoluminescence quantum efficiency (PLQY) was measured for the five complexes in three different states: solution, powder, and PMMA matrix, using the absolute method:^[38]^

All five complexes show excellent values (Table 1) of photoluminescence quantum efficiency (PLQY) of the studied complexes exhibit a range of values depending on the medium: from 52 to 55 % in DCM solution, from 32 to 72 % in solid‐state, and from 70 to 85 % in PMMA matrix. These results can be attributed to the presence of chromophores (TTA and Phenanthroline ligands) that directly participate in the sensitization of the Eu ion in Eu(TTA)_3_L_1‐5_ complexes. Xu et al.^[39]^ recommend that the improvement of the photoluminescence properties of europium complexes can be attended by the introduction of chromophores through steric effects and decrease intramolecular energy transfer efficiently.^[39]^ Results from Table 1 reveals that the photoluminescence quantum yield (PLQY) values of complexes incorporated in PMMA are considerably higher than those in the solid‐state and in solution. This phenomenon can be attributed to the introduction of the PMMA matrix, which plays a key role in providing a rigid support system for the complexes. As a result, the negative effects caused by oscillators at different energy levels (such as C−H, C−C or C−N) and intermolecular collisions between complexes are effectively mitigated. Ultimately, this mechanism contributes significantly to the protection of complexes against photodegradation.^[40]^

**Table 1:** Photoluminescence properties of the complexes in solution, solid‐state and matrix at 298 K.

	CH_2_Cl_2_ conc. 4.7×10^–4^ mol L^–1^			Powder			PMMA (5 % wt)		
	λ_ex_ (nm)	λ_em_ (nm)	Φ_em_ (%)	λ_ex_ (nm)	λ_em_ (nm)	Φ_em_ (%)	λ_ex_ (nm)	λ_em_ (nm)	Φ_em_ (%)
Eu (TTA)_3_ L_1_	371	614	53.68	369	614	72.07	381	614	70.99
Eu (TTA)_3_ L_2_	368	614	54.28	382	615	52.64	371	614	70.64
Eu (TTA)_3_ L_3_	369	614	52.10	374	614	32.14	370	614	74.56
Eu (TTA)_3_ L_4_	369	614	53.66	370	614	55.63	373	614	79.24
Eu (TTA)_3_ L_5_	369	614	55.56	370	614	62.45	374	614	85.47

### Photostability results

The stability of europium complexes is of utmost importance in many applications that exploit these luminescent materials, particularly in the fields of lighting and optoelectronic devices. In order to ensure their long‐term effectiveness, it is crucial to evaluate the stability of these complexes under various conditions, including darkness, exposure to indoor, and outdoor atmosphere. This assessment provides invaluable insights into their durability and performance across diverse environments. Notably, the stability of europium complexes incorporated within a polymer matrix under different environmental conditions has not been explored in the literature to date. Thus, our investigation in this area represents a significant milestone, setting a precedent for future studies in this research field.

Examining the stability of these systems holds immense significance as it offers a deeper understanding of how these complexes behave and endure under specific real conditions, such as humidity, light exposure, and other environmental factors. This knowledge empowers us to better assess their potential applications and optimize their performance accordingly. Furthermore, our pioneering study paves the way for novel research perspectives focusing on the stability of europium complexes in polymeric materials, thereby creating opportunities for the development of robust and long‐lasting luminescent systems.

In the present study, we conducted three photostability tests on films containing five europium complexes embedded in a PMMA polymer matrix. The objective was to assess the photostability of these complexes under different environmental conditions. The samples were exposed to darkness, indoor conditions, and outdoor exposure for a total duration of 1200 hours. Regular measurements were taken every 400 hours to monitor any changes or undesirable effects that may have occurred during the testing period.

**Figure 11:**
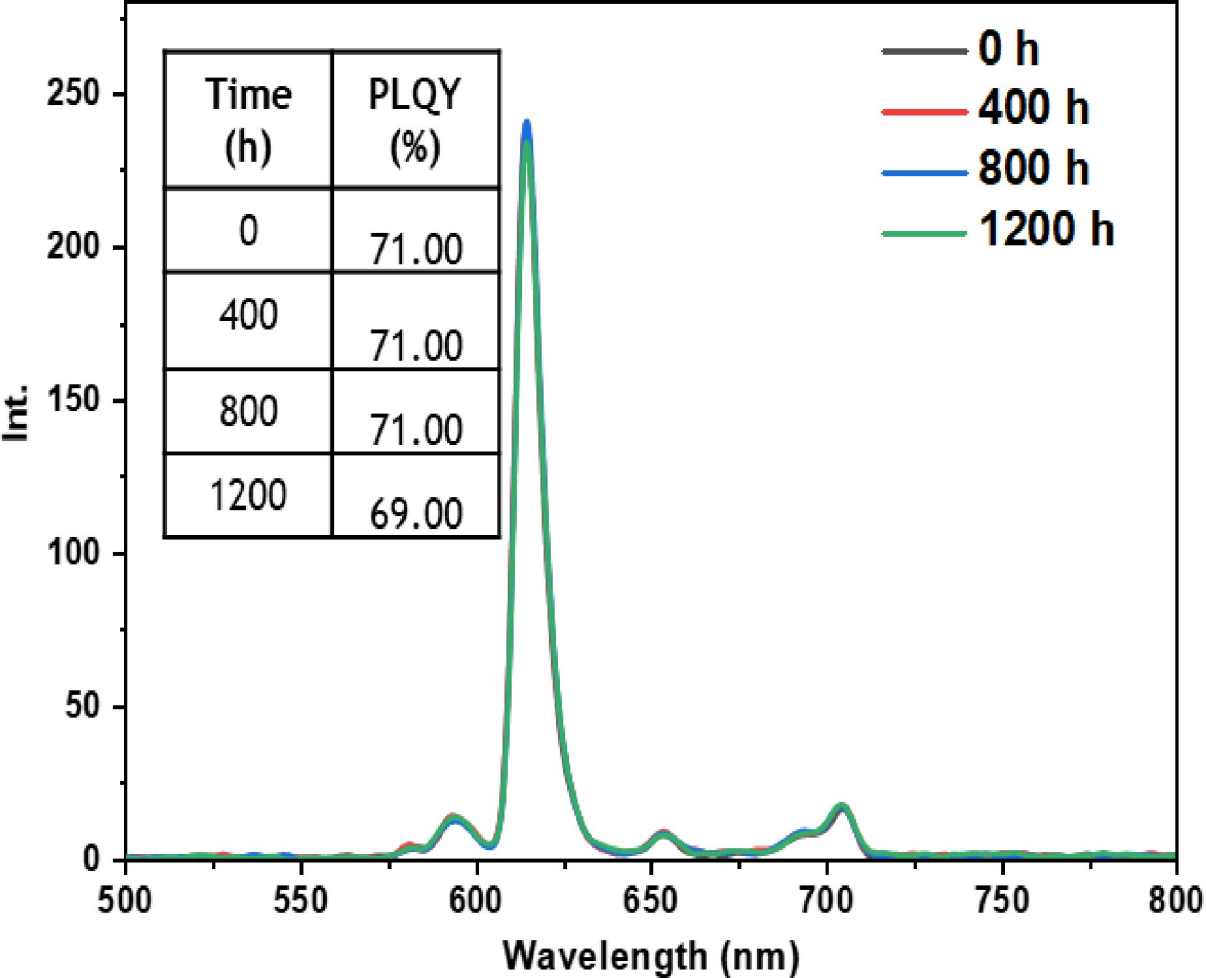
Emission intensity spectra and the PLQY value of the PMMA film doped **Eu(TTA)_3_ L_1_** complex T=0 h and T=1200 h under dark conditions.

**Figure 12:**
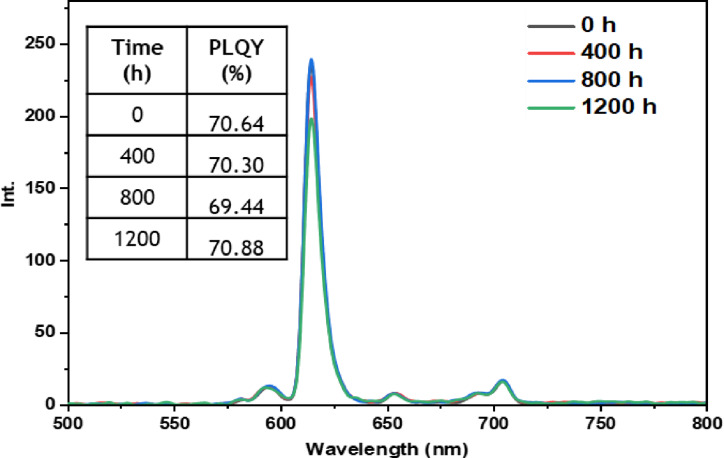
Emission intensity spectra and the PLQY value of the PMMA film doped **Eu(TTA)_3_ L_2_** complex T=0 h and T=1200 h under dark conditions.

**Figure 13:**
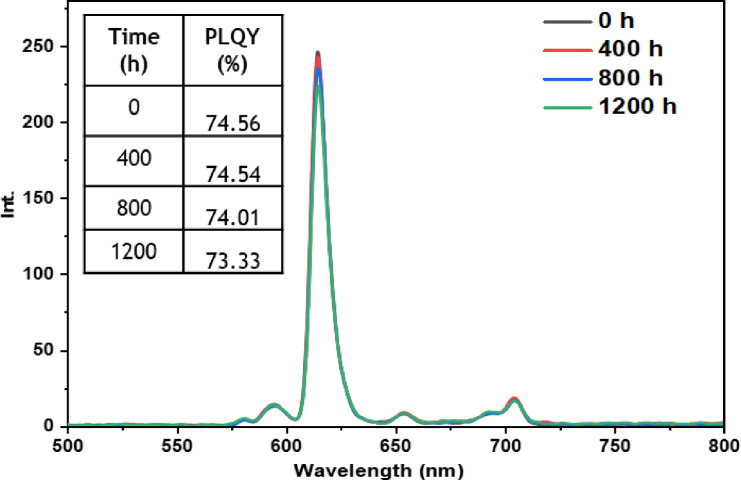
Emission intensity spectra and the PLQY value of the PMMA film doped **Eu(TTA)_3_ L_3_** complex between T=0 h and T=1200 h under dark conditions.

**Figure 14:**
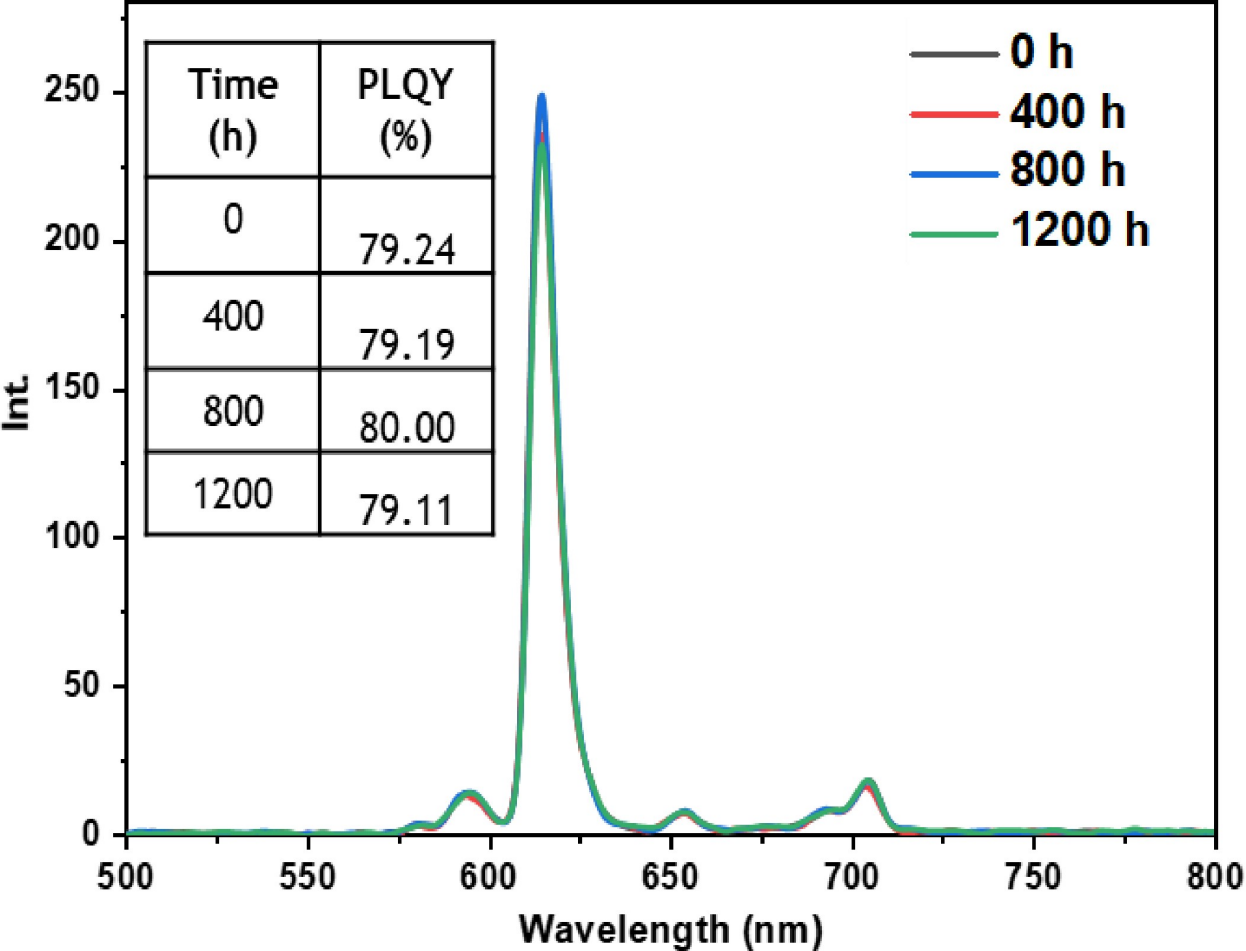
Emission intensity spectra and the PLQY value of the PMMA film doped **Eu(TTA)_3_ L_4_** complex between T=0 h and T=1200 h under dark conditions.

**Figure 15:**
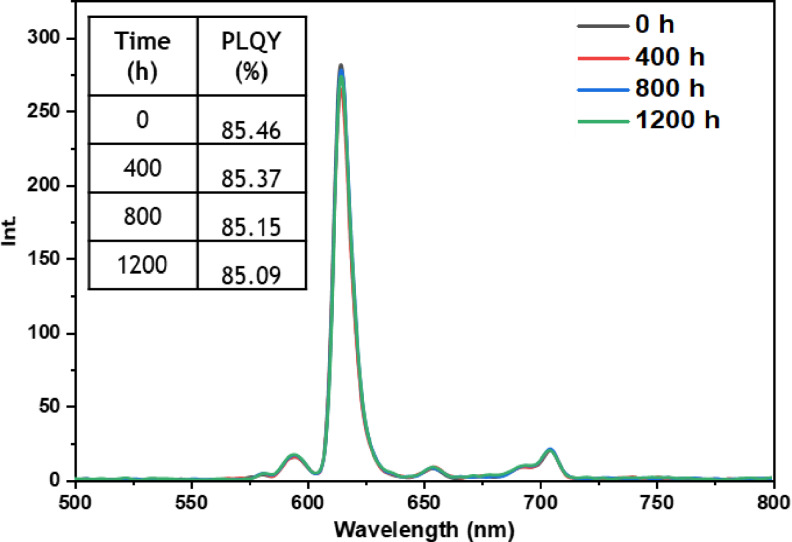
Emission intensity spectra and the PLQY value of the PMMA film doped **Eu(TTA)_3_ L_5_** complex between T=0 h and T=1200 h under dark conditions.

**Figure 16:**
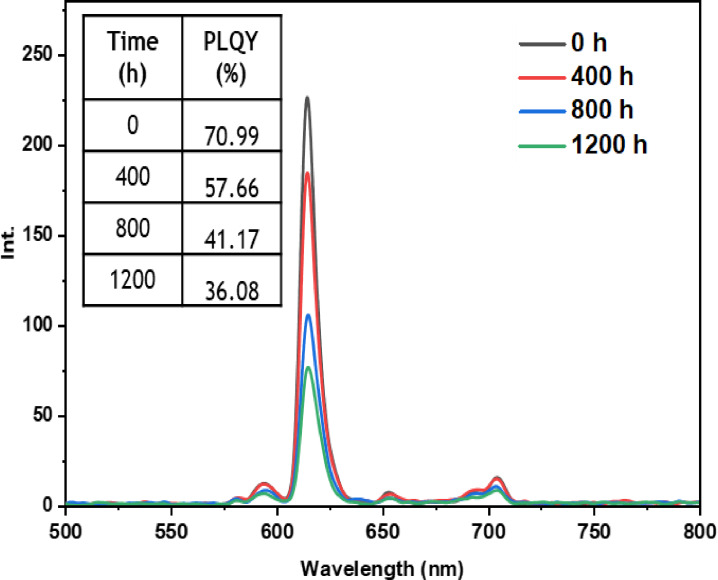
Emission intensity spectra and the PLQY value of the PMMA film doped **Eu(TTA)_3_L_1_** complex between T=0 h and T=1200 h under indoor conditions.

**Figure 17:**
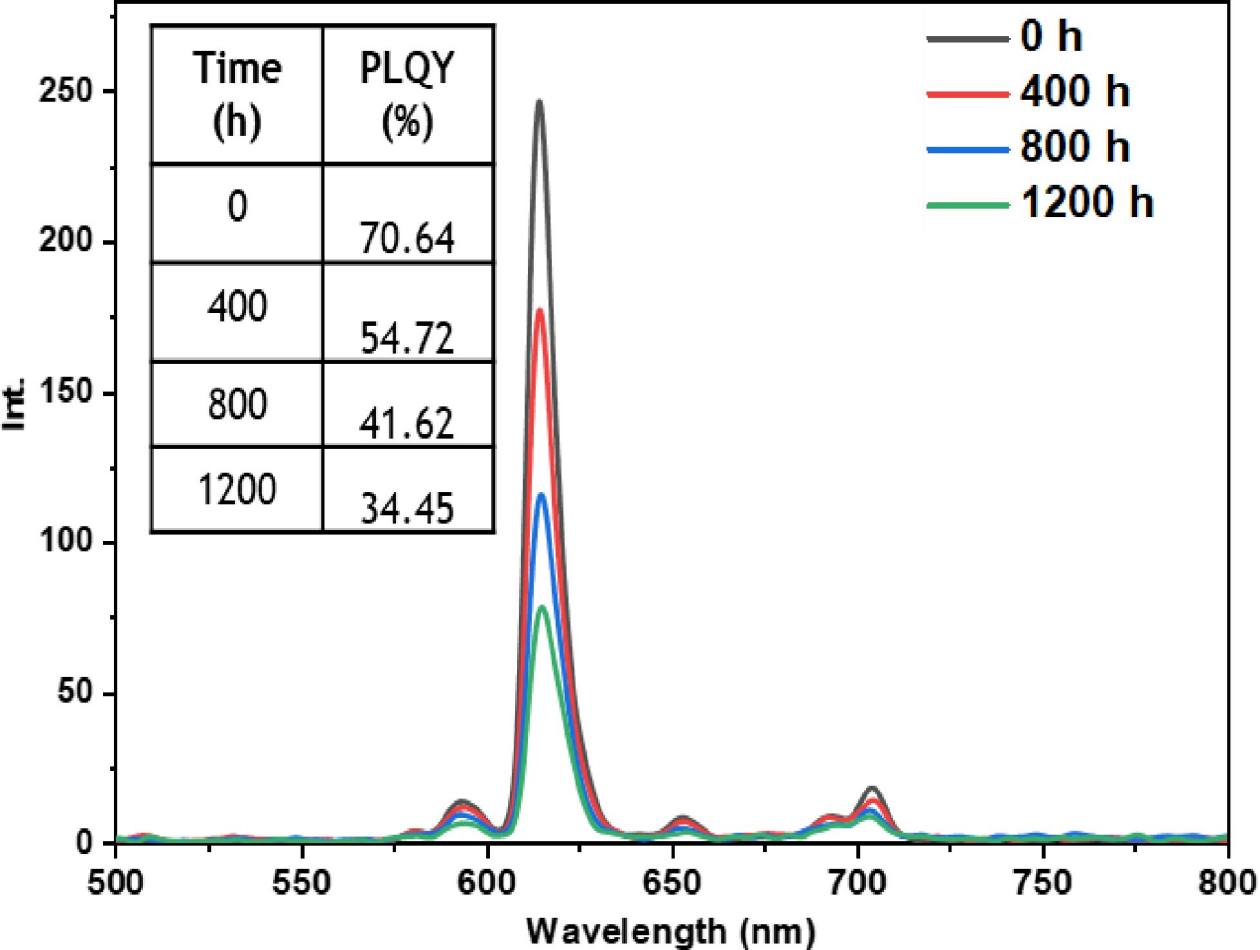
Emission intensity spectra and the PLQY value of the PMMA film doped **Eu(TTA)_3_L_2_** complex between T=0 h and T=1200 h under indoor conditions.

**Figure 18:**
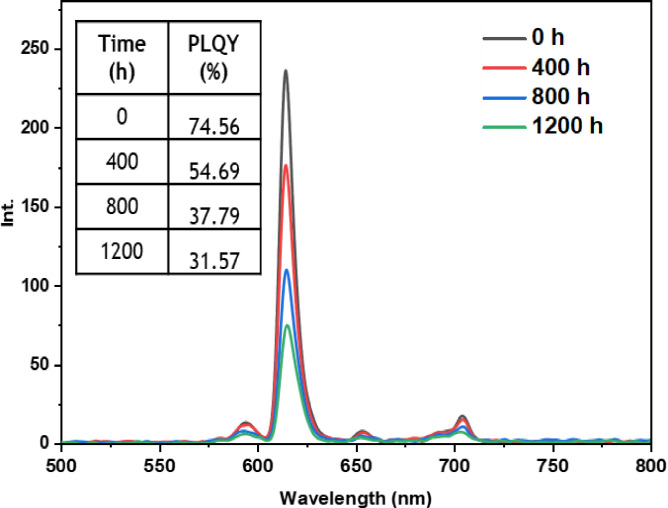
Emission intensity spectra and the PLQY value of the PMMA film doped **Eu(TTA)_3_L_3_** complex between T=0 h and T=1200 h under indoor conditions.

**Figure 19:**
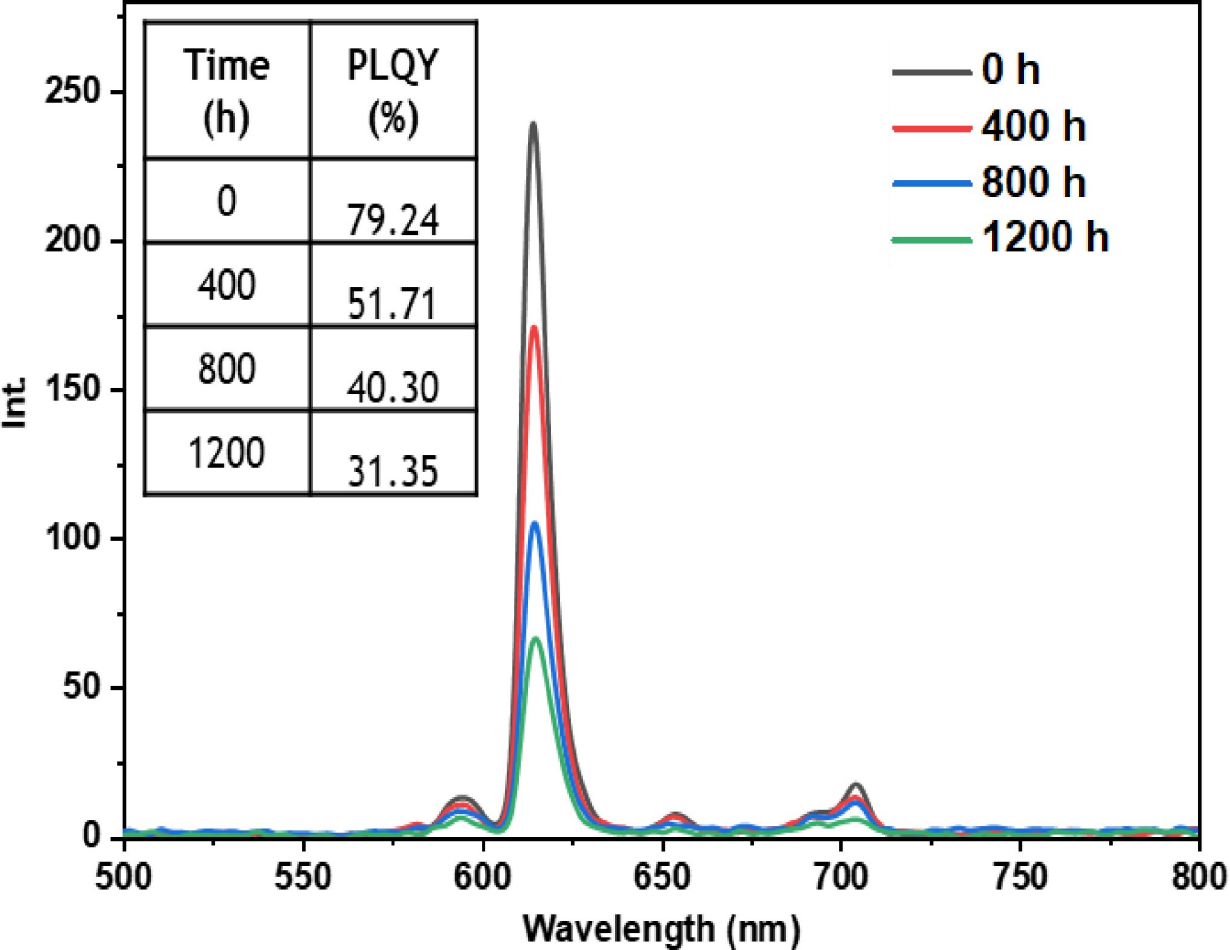
Emission intensity spectra and the PLQY value of the PMMA film doped **Eu(TTA)_3_L_4_** complex between T=0 h and T=1200 h under indoor conditions.

**Figure 20:**
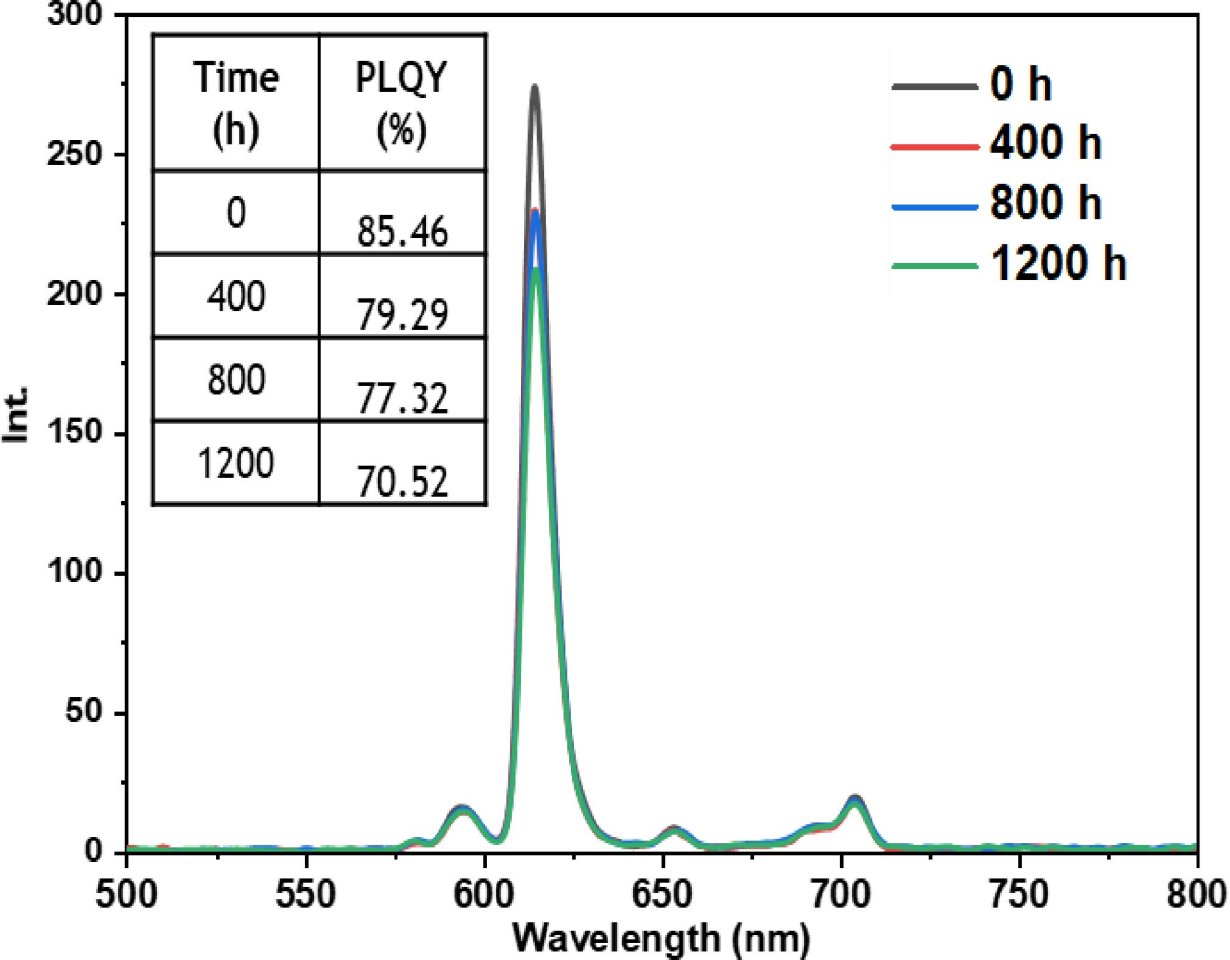
Emission intensity spectra and the PLQY value of the PMMA film doped **Eu(TTA)_3_L_5_** complex between T=0 h and T=1200 h under indoor conditions.

• **Dark condition test**: The dark test was conducted to evaluate the inherent stability of the europium complexes without the influence of external environmental factors. By isolating the samples from light, we aimed to observe if any undesirable reactions or significant changes in the luminescent properties of the complexes occurred over time. The results of this test, as depicted in Figures 11, 12, 13, 14, and 15, indicate that films incorporating the five complexes are generally stable, and demonstrate the ability to retain their photophysical properties over extended periods when not exposed to aggressive environmental conditions and shielded from light. However, it should be noted that the photostability may vary depending on the specific nature of the complex and the ligands used. Some complexes may exhibit increased stability in the dark, while others may be more sensitive to factors such as humidity or oxidation as in the case of the Eu(TTA)_3_L_2_ complex which shows a slight decrease in the intensity of peak.

• **Indoor condition test**: On the other hand, when films containing europium complexes are exposed to the indoor environment, they may be influenced by factors such as relative humidity, ambient temperature, and sunlight. These environmental conditions can lead to the degradation of the complexes and the loss of their luminescent activity. The emission wavelengths and corresponding quantum yields (PLQY) of the five europium complexes incorporated in the PMMA matrix are illustrated in Figures 16, 17, 18, 19, and 20. The results clearly demonstrate that all films based on these five complexes underwent a reduction in the intensity of their emission peak, leading to a decrease in the PLQY value. Notably, the first four complexes (Eu(TTA)_3_L_1‐4_) exhibited similar photostability behavior, displaying a significant decrease in intensity over time as well as PLQY values from around 70.64–79.24 % at T=0 h to 31.35‐36.08 % at T=1200 h.

This can be explained by the fact that some europium complexes can exhibit a certain sensitivity to moisture, which can lead to undesirable reactions, such as hydrolysis of chemical bonds in the complex,^[41]^ especially as the acrylate polymer matrix family has a certain ability to gas and moisture permeation.^[42]^ This can lead to a reduction in the luminescent activity of the complex and an alteration in its chemical stability.

Significantly, the thin film containing the Eu(TTA)_3_L_5_ complex demonstrated exceptional stability when subjected to indoor exposure over a duration of 1200 hours, surpassing the other complexes in terms of resilience. Despite a slight decrease of approximately 17.5 % in its PLQY compared to its initial value at T=0 h, this slight reduction can be attributed to the advantageous presence of additional methyl groups within the complex. These methyl groups enhance its hydrophobicity, reinforcing the complex‘s chemical stability and effectively preserving its luminescent properties even after prolonged exposure.

**Figure 21:**
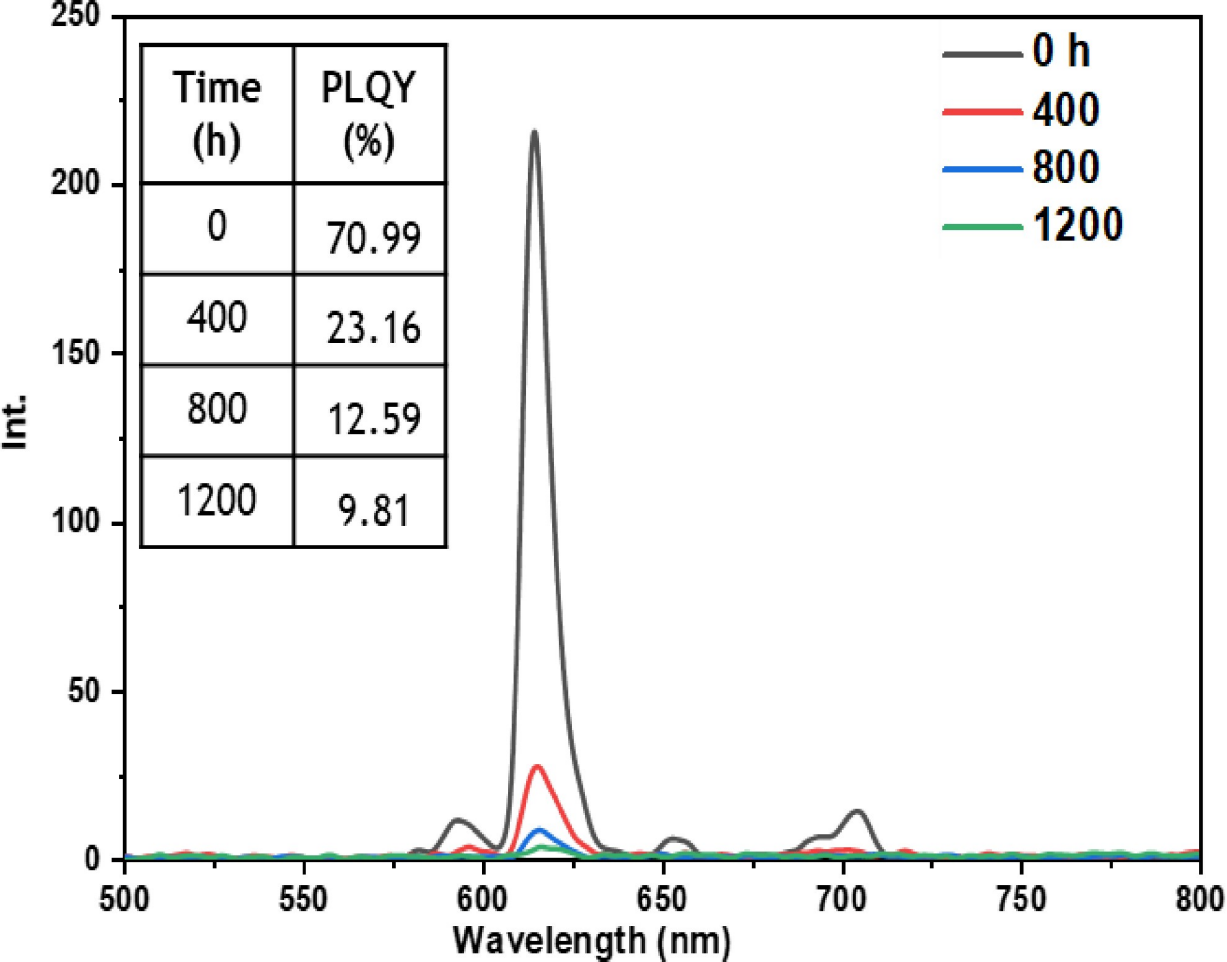
Emission intensity spectra and the PLQY value of the PMMA film doped **Eu(TTA)_3_L_1_** complex between T=0 h and T=1200 h under outdoor conditions.

**Figure 22:**
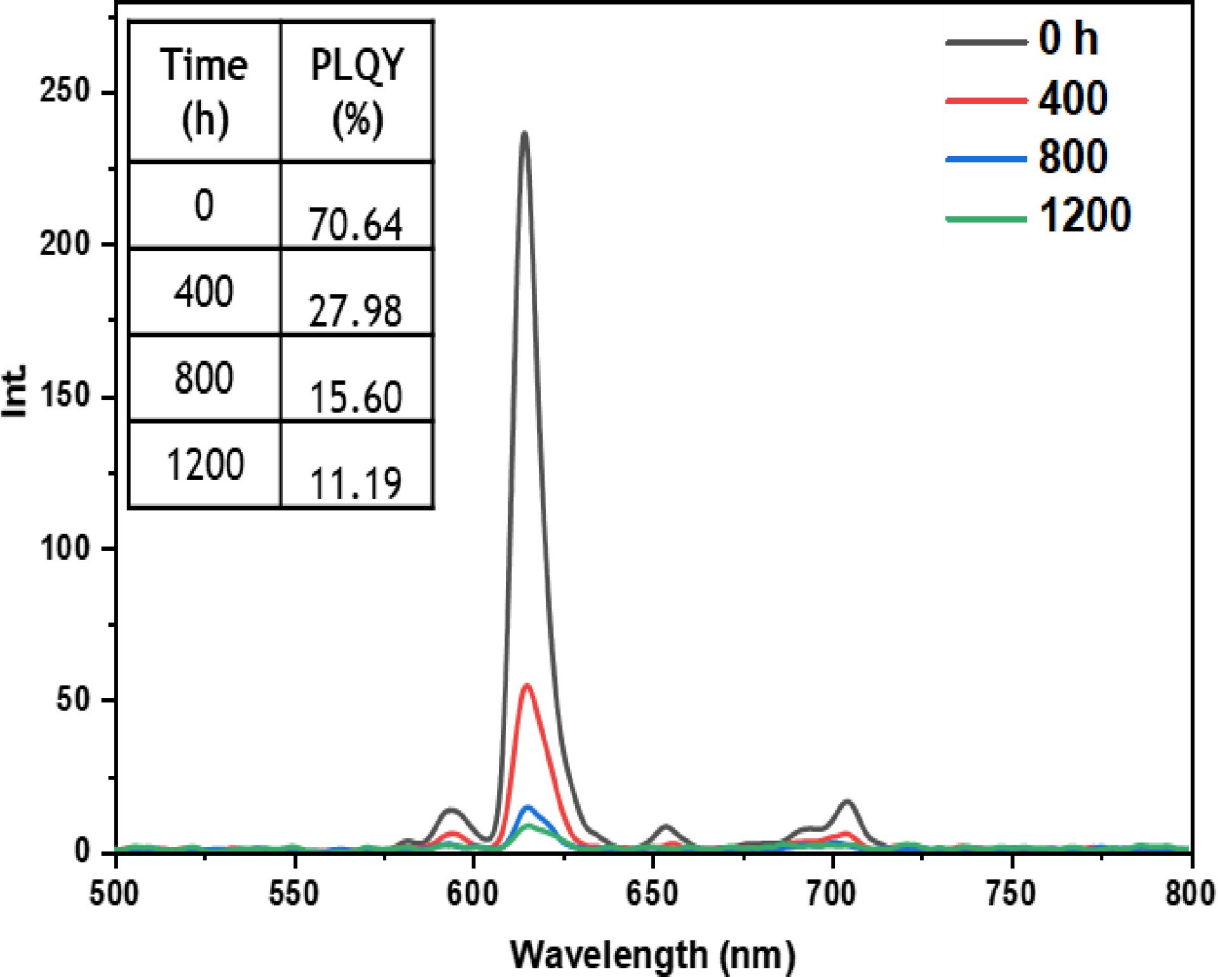
Emission intensity spectra and the PLQY value of the PMMA film doped **Eu(TTA)_3_L_2_** complex between T=0 h and T=1200 h under outdoor conditions.

**Figure 23:**
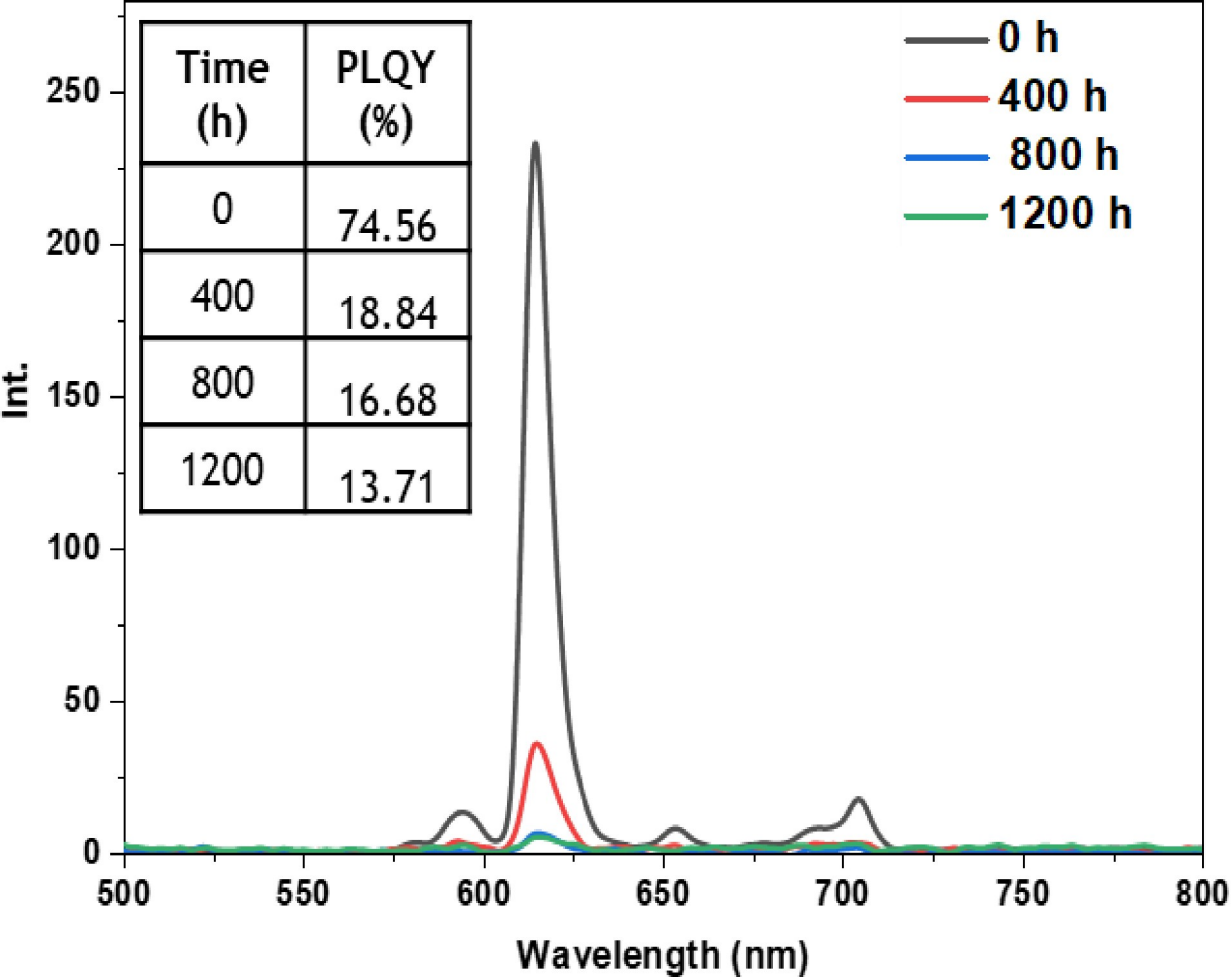
Emission intensity spectra and the PLQY value of the PMMA film doped **Eu(TTA)_3_L_3_** complex between T=0 h and T=1200 h under outdoor conditions.

**Figure 24:**
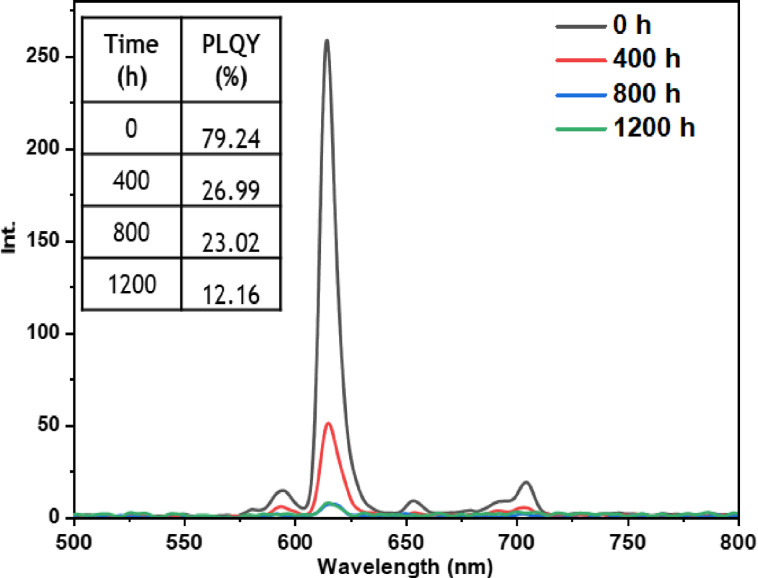
Emission intensity spectra and the PLQY value of the PMMA film doped **Eu(TTA)_3_L_4_** complex between T=0 h and T=1200 h under outdoor conditions.

**Figure 25:**
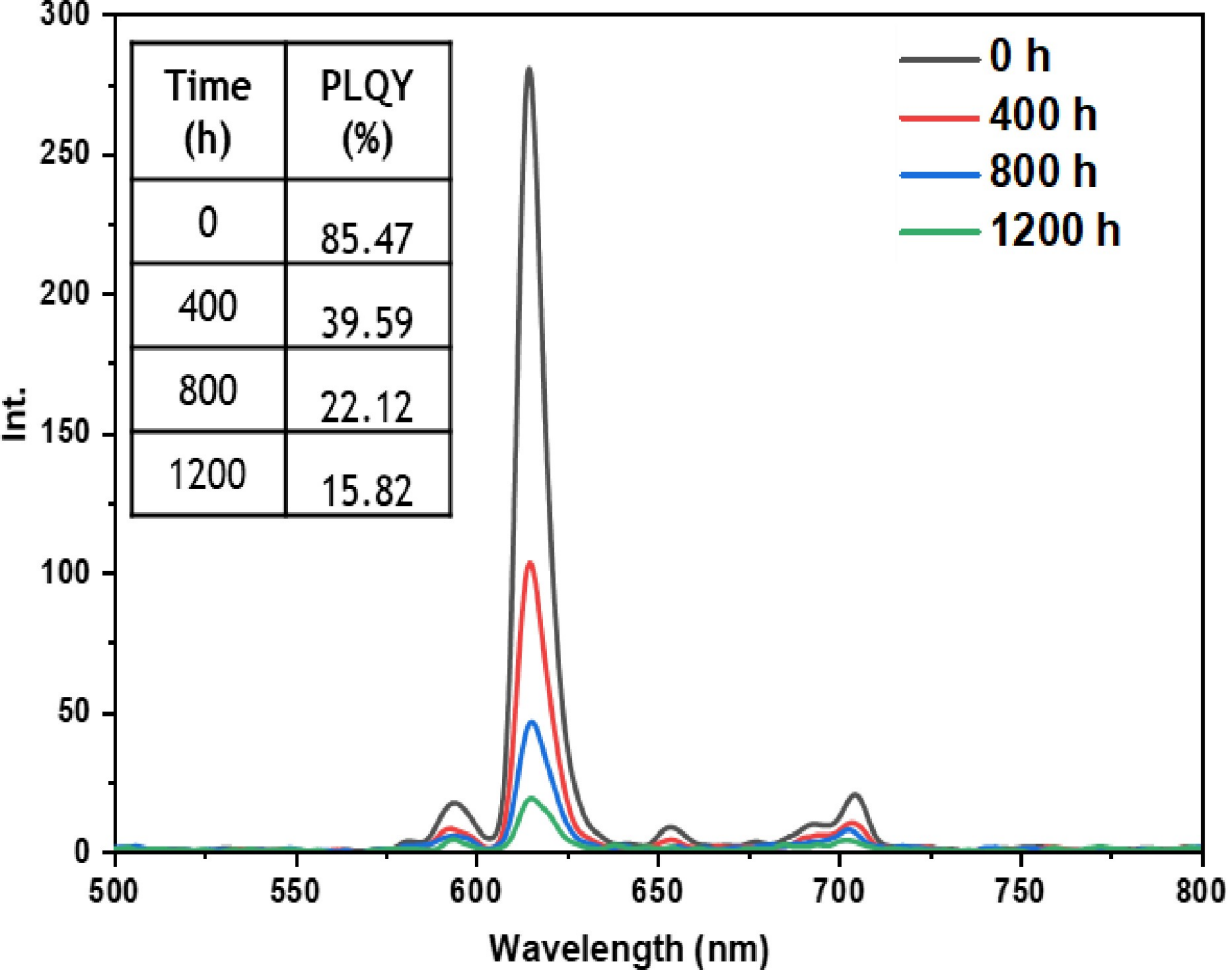
Emission intensity spectra and the PLQY value of the PMMA film doped **Eu(TTA)_3_L_5_** complex between T=0 h and T=1200 h under outdoor conditions.

• **Outdoor condition test**: The outdoor exposure test aimed to simulate real‐life environmental conditions encountered by luminescent materials, assessing the stability and performance of europium complexes in practical applications. These conditions encompassed direct exposure to outdoor temperature, humidity, and sunlight, which are known to affect material performance. Figures 21, 22, 23, 24, and 25 illustrate the results obtained for films based on the complexes. Remarkably, all europium complexes exhibited a significant reduction in luminescence after 1200 hours of exposure, which can be attributed to the combined effect of various climatic factors such as temperature, humidity, and solar radiation. Furthermore, it is worth mentioning that among the tested complexes, the Eu(TTA)_3_L_5_ complex demonstrated superior photoluminescent stability compared to the other complexes, even under these challenging conditions. This finding underscores the potential of the Eu(TTA)_3_L_5_ complex for maintaining its photoluminescent properties in demanding environments.

**Figure 26:**
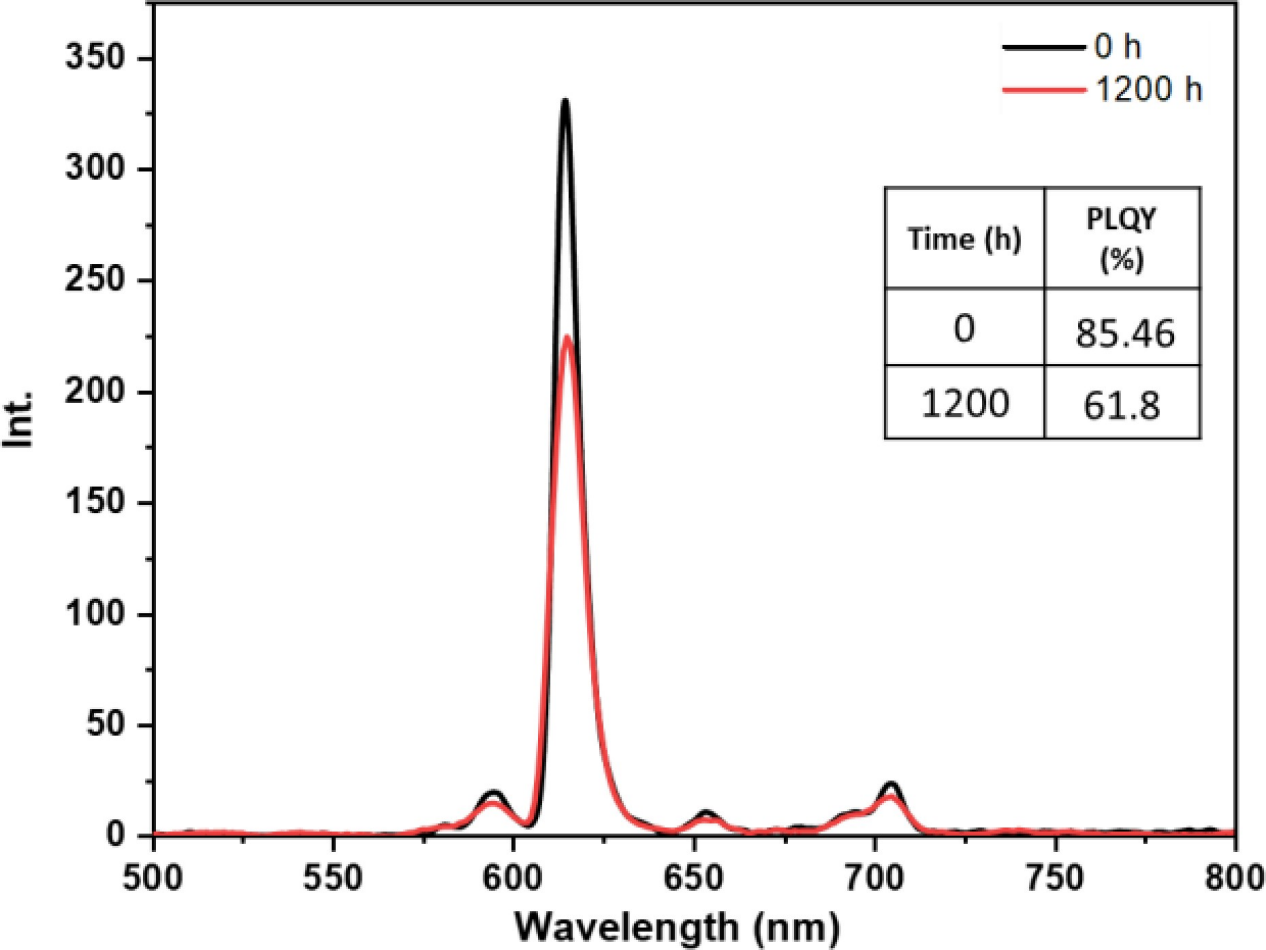
Emission spectra of PMMA films based on Eu(TTA)_3_L_5_ complex at T=0 h and T=1200 h under light soaking.

**Figure 27:**
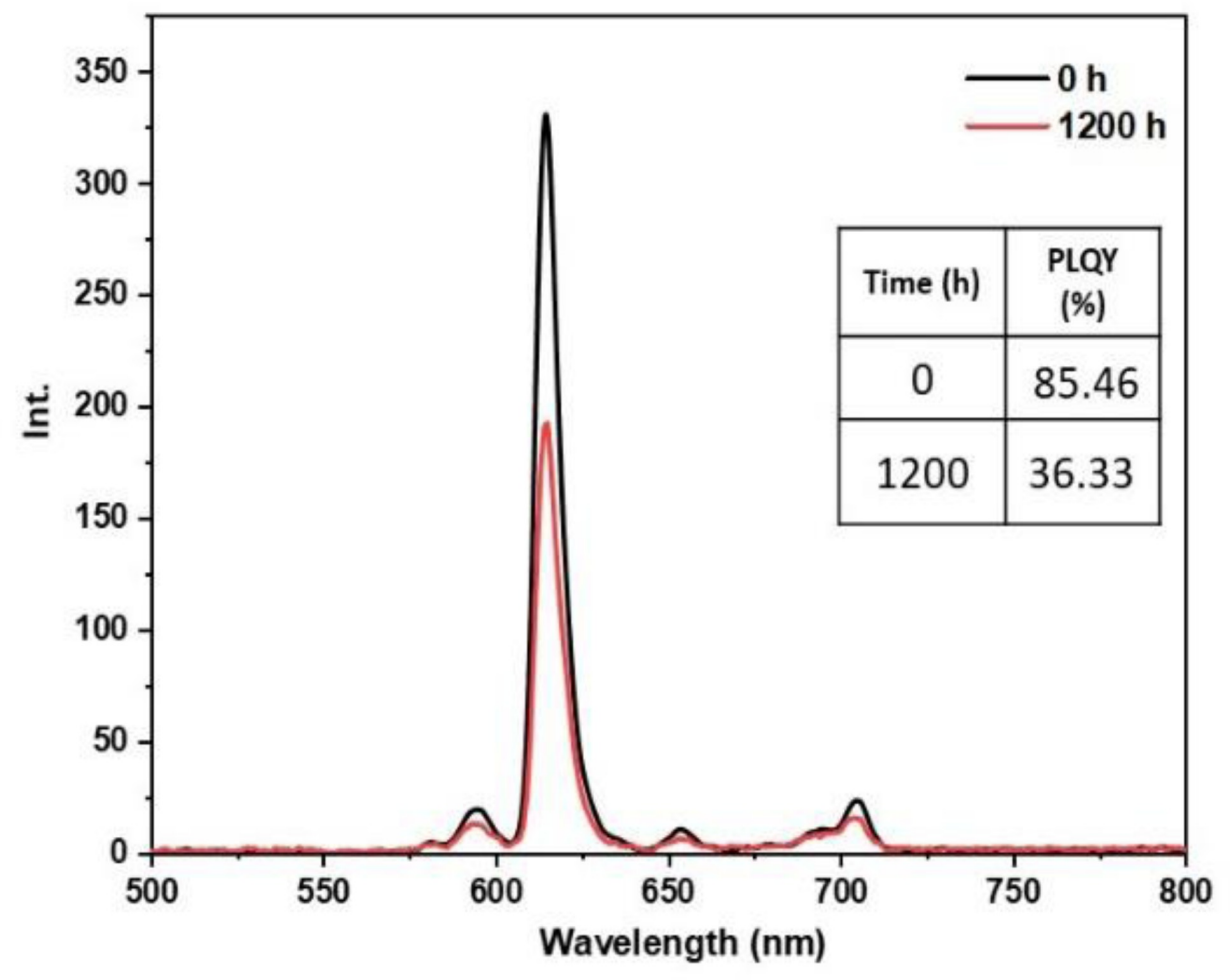
Emission spectra of PMMA films based on Eu(TTA)_3_L_5_ complex at T=0 h and T=1200 h under humidity conditions.

A comprehensive study was conducted to better understand the parameter that most influences the photoluminescent stability of europium complexes incorporated in PMMA. The Eu(TTA)_3_L_5_ complex‐containing sample was subjected to separate investigations under high humidity and light exposure for a duration of 1200 hours. The sample was tested in a humid environment (85 % relative humidity) at a temperature of 40 °C to generate water vapor in a desiccator. Additionally, it was exposed to continuous light generated by a lamp inside another vacuum desiccator throughout the test. The results of emission intensity and PLQY values are summarized in Figures 26 and 27. According to these findings, both humidity and light had a significant effect on the photostability of the complex. The PLQY decreased by approximately 38 % under light exposure and about 64 % under humidity exposure, indicating that humidity had a more pronounced impact on the stability of the complex compared to light exposure. However, the complex exhibited greater resilience when exposed to weather conditions, which strengthens the hypothesis of a synergistic effect of humidity and light on the photoluminescent stability of the complex.

- **Under UV irradiation test**: As previously mentioned, the photostability of the film doped with complexes is of crucial importance in practical applications of optoelectronic devices. In order to assess this photostability, the Eu(TTA)₃L_5_ complex‐based film was investigated under 365 nm UV light. The luminescent emission intensity of the Eu(TTA)₃L_5_ film was recorded at different exposure intervals, including after 0, 1, 3 and 24 hours (see Figure 28
  **Figure 28:**
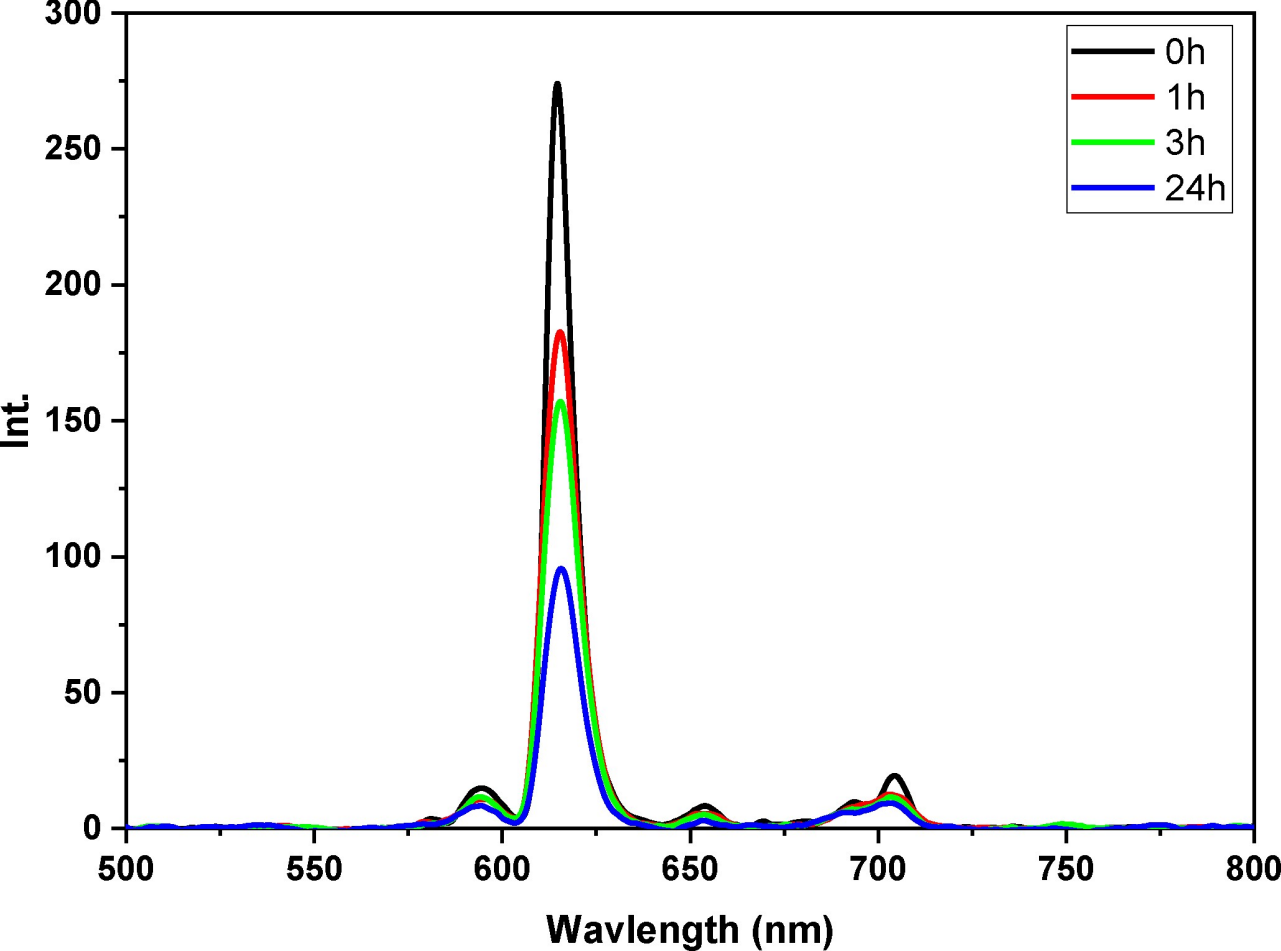
Emission spectra of PMMA films based on Eu(TTA)_3_L_5_ complex under UV irradiation.
  ). During the first few hours of exposure, a slight loss of emission intensity and a decrease in photoluminescence quantum yield (PLQY) were observed for the thin film. In particular, after just one hour of irradiation, a loss of intensity was already observed. This trend became more pronounced after three hours′ exposure, with a loss of in both emission intensity and PLQY (around 10 %). The degradation of photostability intensified after a 24‐hour exposure, marked by a significant decrease in emission intensity, reaching around 60 %. Similarly, the PLQY value showed a reduction of around 30 % compared with its initial value. It has been observed that TTA (thenoyltrifluoroacetone) is highly efficient in amplifying the luminescence of Eu(III) however, its effectiveness diminishes notably upon exposure to the UV light employed for Eu(III) excitation. ^[43]^ These results highlight the potential challenges associated with the photostability of Eu(TTA)₃L_5_ film under prolonged UV irradiation, underscoring the importance of taking this factor into account in the design and practical application of optoelectronic devices based on these complexes.

## Conclusions

The findings demonstrated that the film incorporating the europium complex with the phenanthroline ligand substituted with a higher number of methyl groups (Eu(TTA)_3_L_5_) exhibited superior stability under all exposure conditions, demonstrating increased resistance to degradation compared to the other complexes studied.

Furthermore, this study highlighted the potential for chemical modification of phenanthroline ligands to enhance the stability of these complexes in films. This improved stability confers great promise on these complexes as candidates for future optoelectronic devices, including luminescent solar concentrators. The results obtained thus contribute to a deeper understanding of the design and development of stable luminescent materials for advanced applications.

## Experimental Section:

### Essential Experimental Procedures/Data Reagents and materials

Potassium tert‐butoxide (KOtBu), 2‐Thenoyltrifluoroacetone (TTA), 10‐phenanthroline (L_1_), 5‐Methyl‐1,10‐phenanthroline (L_2_), 5,6‐Dimethyl‐1,10‐phenanthroline (L_3_), 4,7‐Dimethyl‐1,10‐phenanthroline (L_4_) and 3,4,7,8‐Tetramethyl‐1,10‐phenanthroline (97 %) (L_5_), EuCl_3_,6H_2_O, and Poly(methyl methacrylate) (PMMA) were procured from Sigma‐Aldrich. Dichloromethane (DCM), Ethanol (EtOH) were obtained from Merck and distilled water was used for all the experiments. H_2_O and ethanol (for analysis, Sigma‐Aldrich) were used without further purification.

### Synthesis protocols

The complexes Eu(TTA)_3_L_1_, Eu(TTA)_3_L_2_, Eu(TTA)_3_L_3_, Eu(TTA)_3_L_4_ and Eu(TTA)_3_L_5_ were synthesized according to the processes in the literature:^[7]^ TTA (3 mmol) and KOtBu (3 mmol) were mixed in ultra‐pure water (5 mL) at 60 °C for 30 mins to form a transparent solution before adding salt of EuCl_3_,6H_2_O (1 mmol). Subsequently, a white precipitate was obtained and dissolved in 3 to 5 mL of ethanol. The mixture was stirred for an extra 30 minutes before adding the one of the equivalent ligands L_1‐5_ (1 mmol) in a solution to form a white precipitate, filtered off, washed with cold water (100 mL), and dried under vacuum for six hours. The final compounds Eu(TTA)_3_ L_1‐5_ were obtained as white crystals in a 65 %, 66 %, 60 %, 73 % and 75 % yield respectively. The complexes were characterized by ^1^H‐NMR, ^19^F‐NMR spectroscopy. Copies of NMR spectra can be found in Supplementary Material (Figure S3, S4, S5, S6 and S7).

### X‐ray crystallography

The selected crystals of Eu(TTA)_3_L_2_ and Eu(TTA)_3_L_3_ were coated with paratone oil and mounted onto the goniometer. The X‐ray crystallographic data were obtained at low temperatures from an XtaLAB Synergy diffractometer (CuKα radiation source) equipped with an Oxford Cryosystem. The structures have been solved with SUPERFLIP^[44]^ and refined by means of least‐square procedures on F^2^ using the PC version of the program CRYSTALS.^[45]^ The scattering factors for all the atoms were used as listed in the International Tables for X‐ray Crystallography. Absorption correction was performed using a multi‐scan procedure. All non‐hydrogen atoms were refined anisotropically. The H atoms were refined with riding constraints. A disorder was found in a thiophene group for both structures. Refinement of the used models led to satisfactory solutions with a 0.5:0.5 and 0.6:0.4 occupancy ratios. The drawing of the molecules was realized with the help of Mercury.

### Crystal data for [Eu(TTA)_3_L_2_]

C_37_H_22_EuF_9_N_2_O_6_S_3_, M=1009.73, triclinic, P‐1, Z=2, a=9.9700(2), b=12.7301(3), c=15.6041(4) Å, α=95.037(2), β=90.932(2), γ=101.974(2)°, V=1928.68(8) Å3, 40060 collected reflections, 8257 unique reflections (Rint=0.057), dcalc=1.739, μCuKα=13.99 mm^−1^. Final R indices: R=0.0683, wR=0.1780 with 5824 reflections ([I>2σ(I)]), 517 parameters, 60 restraints; maximum/minimum residual electron density 2.42/−1.73 e.Å^−3^.

### Crystal data for [Eu(TTA)_3_L_3_]

C_38_H_24_EuF_9_N_2_O_6_S_3_, M=1023.76, orthorhombic, P b c a, Z=8, a=16.5036(1), b=21.4251(1), c=21.8688(1) Å, V=7732.62(7)Å^3^, 231680 collected reflections, 8060 unique reflections (Rint=0.066), dcalc=1.759, μCuKα=13.97 mm^−1^. Final R indices: R=0.0585, wR=0.1579 with 7611 reflections ([I>2σ(I)]), 538 parameters, 20 restraints; maximum/minimum residual electron density 3.22/−2.37 e.Å^−3^.

CCDC‐2165421 and 2283960 contain supplementary crystallographic data for this paper. These data can be obtained free of charge from The Cambridge Crystallographic Data Centre via https://www.ccdc.cam.ac.uk/structures/

Deposition Number(s) 2165421 (for **Eu(TTA)**_**3**_ **L**_**2**_), 2283960 (for **Eu(TTA)**_**3**_ **L**_**3**_) contain(s) the supplementary crystallographic data for this paper. These data are provided free of charge by the joint Cambridge Crystallographic Data Centre and Fachinformationszentrum Karlsruhe Access Structures service.

### NMR measurements

^1^H‐NMR: Nuclear magnetic resonance (NMR) spectra were performed on a 300 MHz PerkinElmer Spectrometer. Chemical shifts δ are counted positive and expressed in ppm relative to tetramethyl silane (TMS). The solvents used were either DMSO or CDCl_3_.

The ^19^F‐NMR spectra were recorded at 600 MHz Bruker spectrometer. TMS was used as an internal standard in ^1^H‐NMR measurement.

### FT‐IR measurements

Fourier transform infrared (FTIR) spectra were determined using a spectrometer (PerkinElmer Spectrum 100) over a range of 450–4000 cm^−1^ with a resolution of 4 cm^−1^ and several scans of 16. KBr pellets were prepared by grinding approximately 1 % of the samples in KBr and compressed into a pellet.

### Thermogravimetric Analysis (TGA)

The thermal stability of the complexes and corresponding films was evaluated through thermogravimetric analysis (TGA) and differential thermogravimetric analysis (DTA). Measurements were carried out over a temperature range from 25 to 700 °C, using a heating rate of 10 °C/min under a nitrogen (N_2_) atmosphere. These analyses were carried out using a TA instrument model SDTQ650.

### UV‐Vis absorption measurement

The ultraviolet‐visible (UV‐vis) spectra were collected on a SHIMADZU spectrophotometer (UV‐2600 UV/VIS), the apparatus contains an integrating sphere equipped with two detectors (photomultiplier tube and an InGaAs detector). The Shimadzu UV‐2600 UV/VIS spectrophotometer can be used to measure absorption or transmission of samples at wavelengths between 185 and 900 nm. The instrument has a linear response down to 1 absorbance unit. Standard (5 ml) and micro (1 ml) quartz cuvettes with a path length of 1 cm are available; the instrument can also be used with disposable plastic cuvettes.

### Photoluminescence measurements

Photoluminescence emission (PL) and excitation spectra are recorded with Jasco Fluorimeter UV/Vis/NIR FP‐8700 single monochromator with dual motorized automatic focusing lenses that vary according to the wavelength to improve energy and sensitivity and 150 W xenon lamp with auto ozone decomposition, automatic compensation of intensity variations, Auto Gain Auto Sensitivity (Auto SCS), with a wavelength range between 300 to 1700 nm, moreover, the quantum yield (PLQY) was obtained with Jasco Fluorimeter UV/Vis/NIR FP‐8700. The PLQY is measured with the absolute method and calculated directly from the equipment with an integration sphere using different holders for liquid, solid, and matrix. Spectra Manager software is used to plot the curves. The absorbance spectrum is recorded with a Jasco V‐770‐UV/VIS/NIR single monochromator with a wavelength range between 190 to 3200 nm.

### Processing of Eu‐PMMA films

The fabrication procedure of Eu‐PMMA films is described as follows: PMMA powder (0.24 g) was dissolved in DCM (20 mL) with stirring, and then 5 prepared complexes of EuTTA)_3_L_1_, Eu(TTA)_3_L_2_, Eu(TTA)_3_L_3_, Eu(TTA)_3_L_4_ and Eu(TTA)_3_L_5_ were added to the mixture separately. The concentration of the complex in PMMA was controlled at 5 wt%. These solutions are placed under stirring for three hours to ensure complete dispersion of the complexes. Then, the prepared solutions are filled into the mold (2 * 3 * 0.2 cm^2^) to prepare five PMMA films based on the five europium complexes. Finally, the mold is placed overnight at room temperature.

### Photostability testing

The photostability testing is carried out in different conditions:

- **Outdoor test**: The prepared samples are mounted in a glass cuvette, to avoid any dust source, and exposed to outdoor conditions of direct irradiation during different times of 0, 400, 800, and 1200 hours.
- **Indoor test**: The prepared samples are placed in indoor conditions of diffuse irradiation at room temperature during different times of 0, 400, 800, and 1200 hours.
- **Dark test**: The prepared samples are kept in a dark space without any light source and at room temperature during different times of 0, 400, 800, and 1200 hours.
- **Under light**: The prepared samples are placed in a vacuum dissector and exposed to direct light for 1200 hours.
- **Under humidity**: The prepared samples are placed in a desiccator containing water and maintained in an oven at 40 °C to achieve a humidity level of 85 % throughout the duration of 1200 hours.
- **Under UV irradiation**: The UV photostability test involves subjecting polymer films containing each of the five distinct complexes to ultraviolet irradiation for a duration of 24 hours, with an operating wavelength of 365 nm. Photophysical property measurements were conducted at intervals of 1, 3, and 24 hours following the initiation of exposure.

## Conflict of interests

The authors declare no conflict of interest.

1

## Supporting information

As a service to our authors and readers, this journal provides supporting information supplied by the authors. Such materials are peer reviewed and may be re‐organized for online delivery, but are not copy‐edited or typeset. Technical support issues arising from supporting information (other than missing files) should be addressed to the authors.

Supporting Information

Supporting Information

Supporting Information

## Data Availability

The data that support the findings of this study are available on request from the corresponding author. The data are not publicly available due to privacy or ethical restrictions.
