# Supplementary material for: Photoluminescence lifetime stability studies of β‐diketonate europium complexes based phenanthroline derivatives in poly(methyl methacrylate) films
Source: ChemistryOpen. 2024 Jan 12;13(5):e202300192. doi: 10.1002/open.202300192 (PMC11095151; doi:10.1002/open.202300192)
Supplement: Supplementary file 2 — Supporting Information [file OPEN-13-e202300192-s003.pdf]

ChemistryOpen

Supporting Information

Photoluminescence lifetime stability studies of β-diketonate europium complexes based phenanthroline derivatives in poly(methyl methacrylate) films

Othmane Essahili,* Mouad Ouafi, Mohamed Ilsouk, Omar Lakbita, Carine Duhayon, Lhassane Mahi, and Omar Moudam*

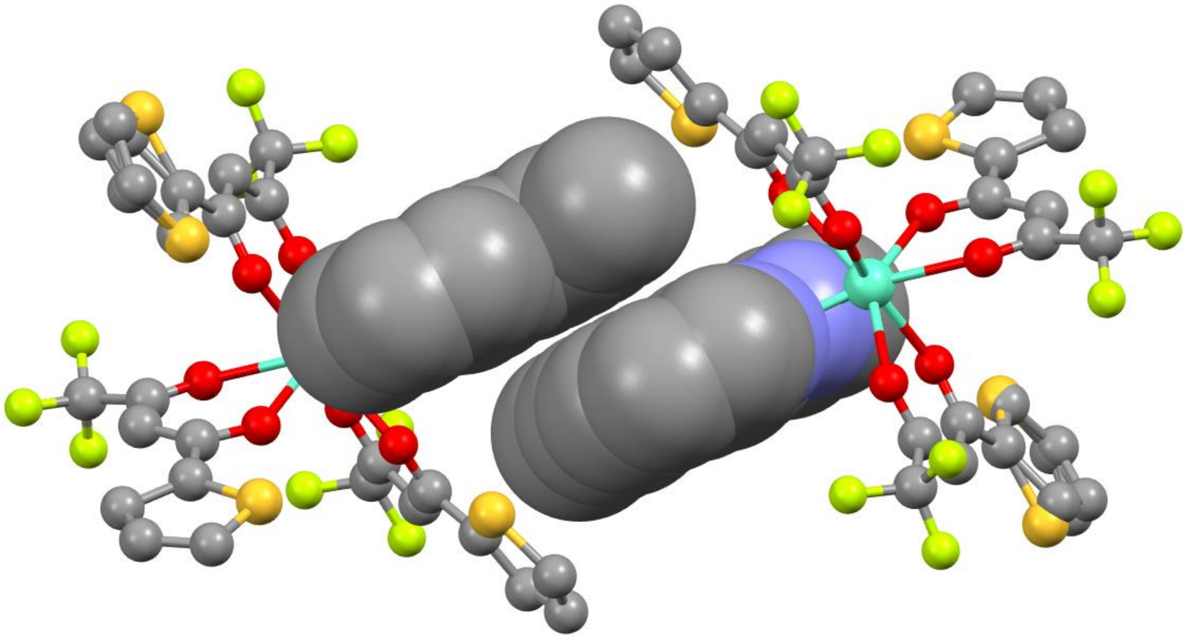

Figure S1. Interaction type between two superposed adjacent phenanthroline ligands for $[Eu(TTA)_3L_2]$ complex

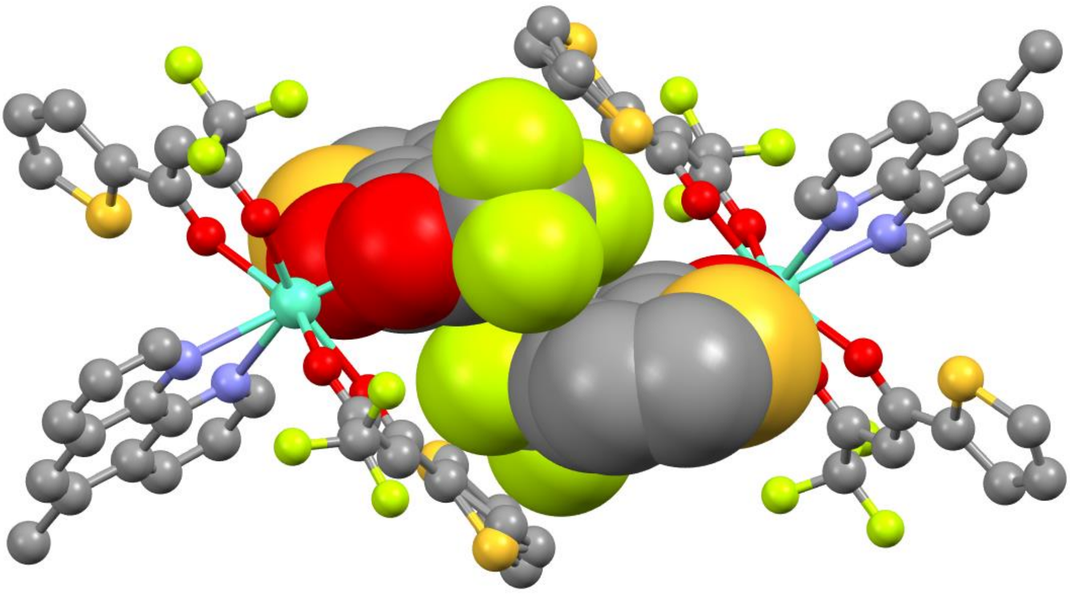

Figure S2. Interaction type between two counterposed adjacent TTA ligands for $[Eu(TTA)_3L_2]$ complex

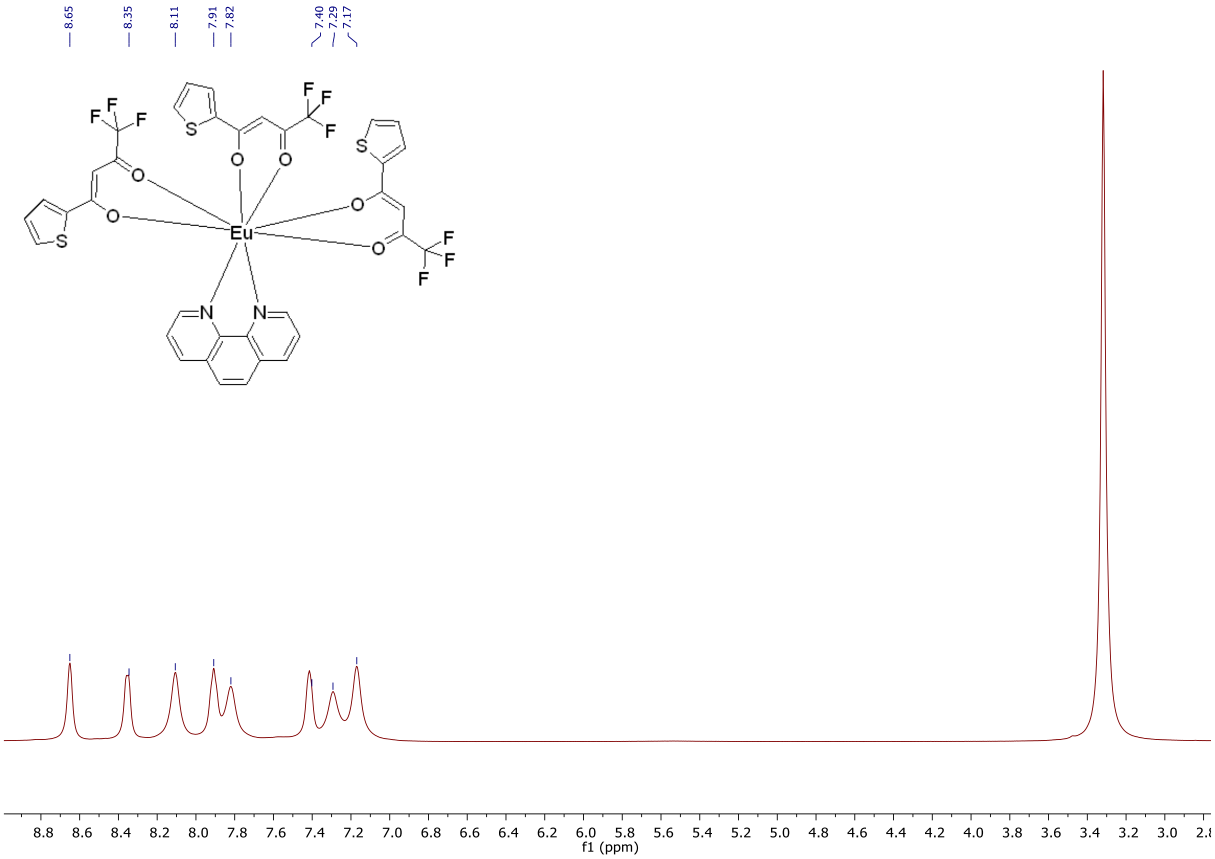

Figure S3: ^{1}H-NMR spectrum of the complex Eu(TTA)$_3$ L$_1$ at 300 MHz in DMSO-D6.

Eu (TTA)$_3$ L$_1$:^{1}H NMR (300 MHz, DMSO-D6) δ 8.65 (s, 2H), 8.35 (s, 2H), 8.11 (s, 2H), 7.91 (s, 3H), 7.82 (s, 3H), 7.40 (s, 2H), 7.29 (s, 3H), 7.17 (s, 3H).

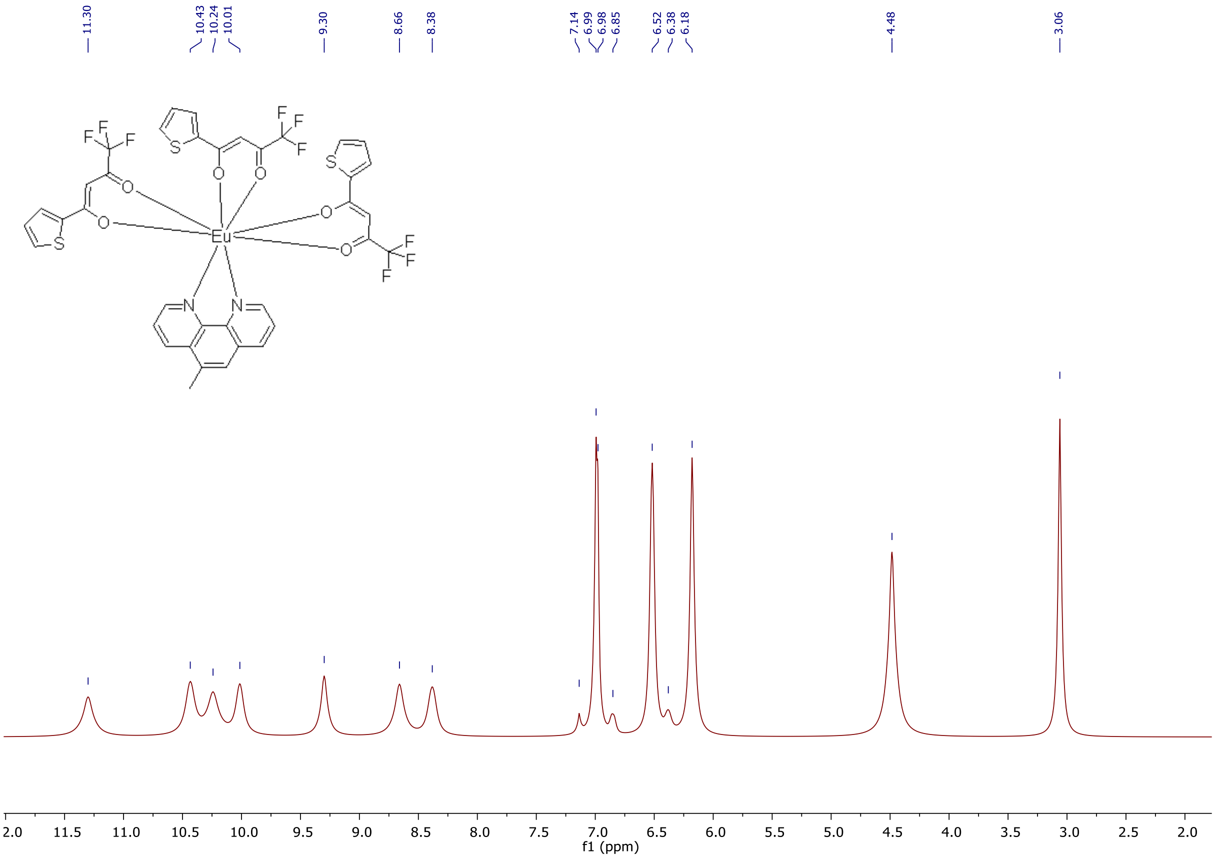

Figure S4: ^{1}H-NMR spectrum of the complex Eu(TTA)$_3$ L$_2$ at 300 MHz in $CDCl_3$.

Eu (TTA)$_3$ L$_2$:^{1}H NMR (300 MHz, $CDCl_3$) δ 11.30 (s, 1H), 10.43 (s, 1H), 10.24 (s, 1H), 10.01(s, 1H), 9.30 (s, 1H), 8.66 (s, 1H), 8.38 (s, 1H), 6.99 (dd, J= 2 Hz, 3H), 6.52 (d, J= 2 Hz, 3H), 6.18 (s, 3H), 4.48 (s, 3H), 3.06 (s, 3H).

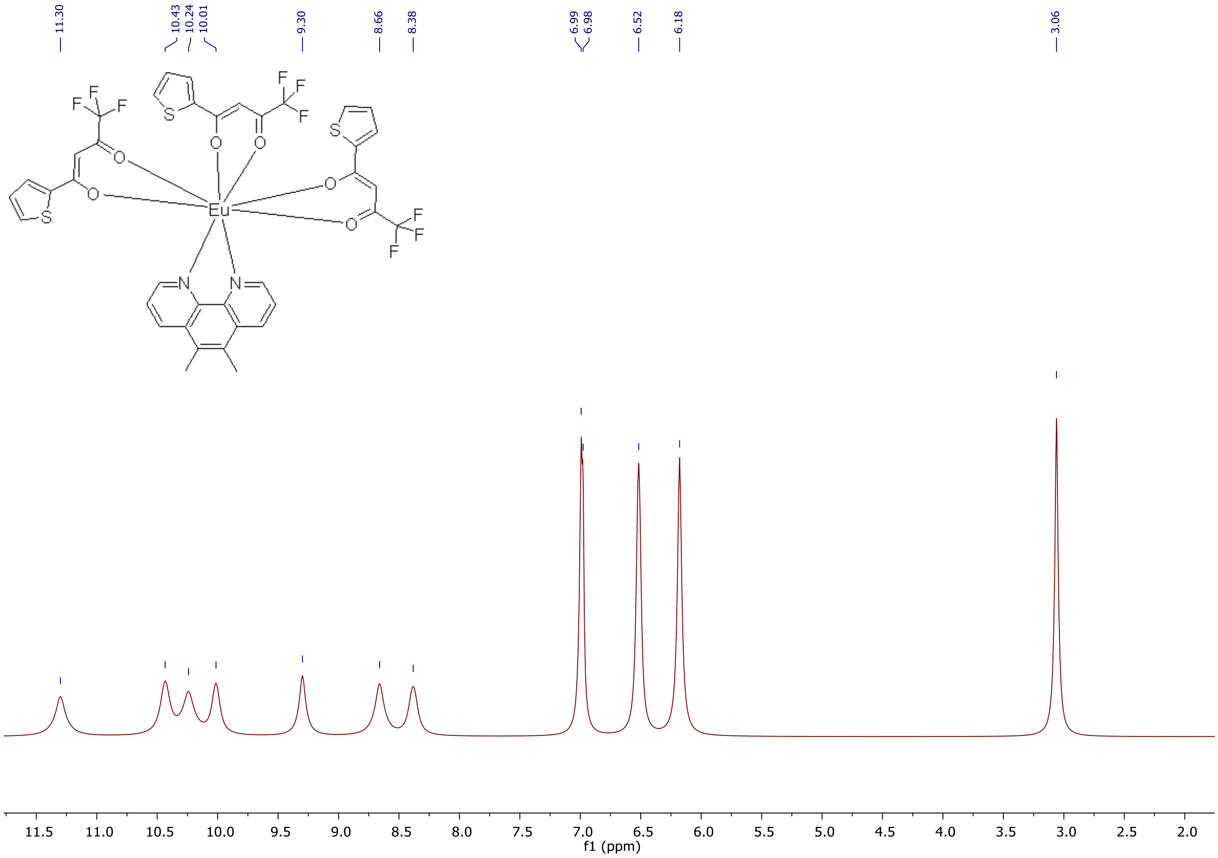

Figure S5: ^{1}H-NMR spectrum of the complex Eu(TTA)$_3$ L$_3$ at 300 MHz in $CDCl_3$.

Eu (TTA)$_3$ L$_3$:^{1}H NMR (300 MHz, $CDCl_3$) δ 11.30 (s, 2H), 10.24 (s, 2H), 9.30 (s, 2H), 8.66 (s, 3H), 6.99 (d, J= 2 Hz, 3H), 6.52 (s, 3H), 6.18 (s, 3H), 3.06 (s, 6H).

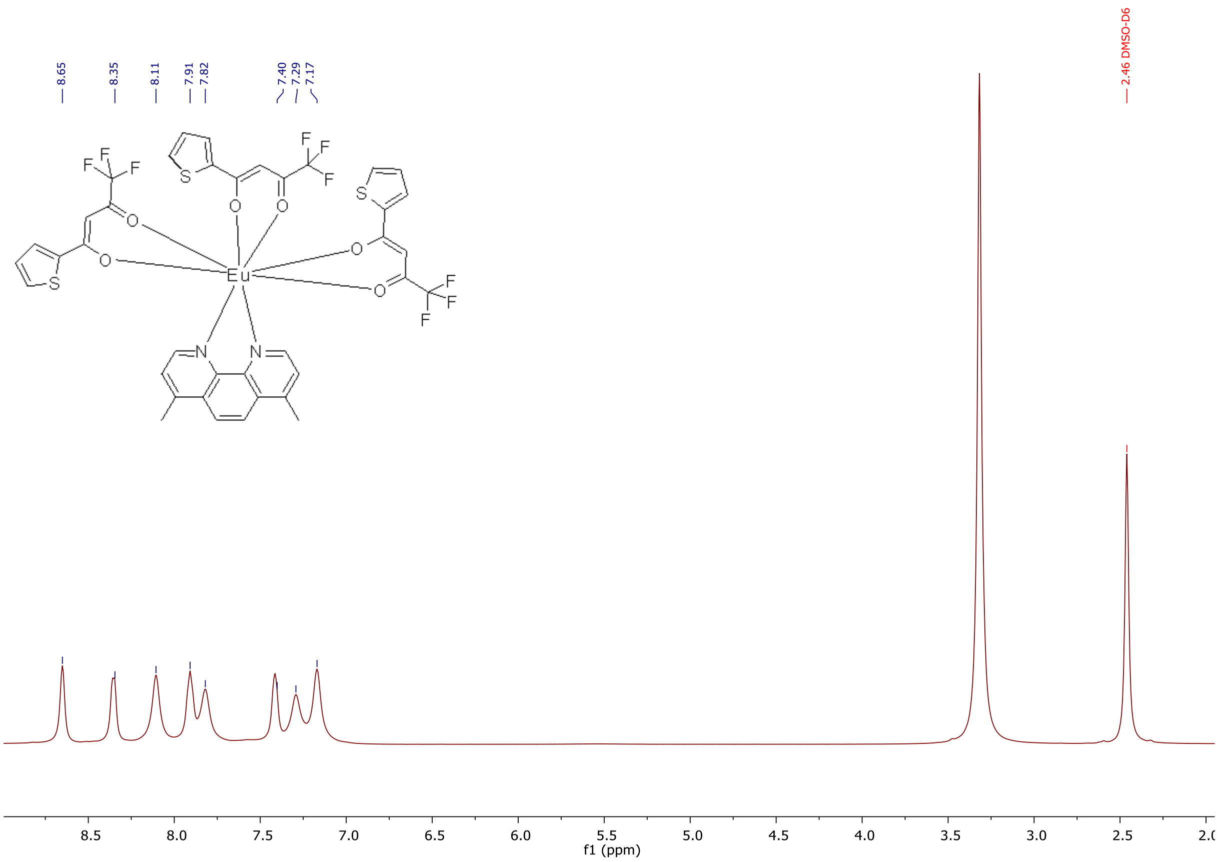

Figure S6: ^{1}H-NMR spectrum of the complex Eu(TTA)$_3$ L$_4$ at 300 MHz in DMSO-D6.

Eu (TTA)$_3$ L$_4$: ^{1}H NMR (500 MHz, DMSO-D6) δ 8.65 (s, 2H), 8.35 (s, 2H) 8.11 (s, 3H), 7.91 (s, 3H), 7.57 (s, 2H), 7.29 (s, 3H), 7.17 (s, 3H), 2.46 (s, 6H).

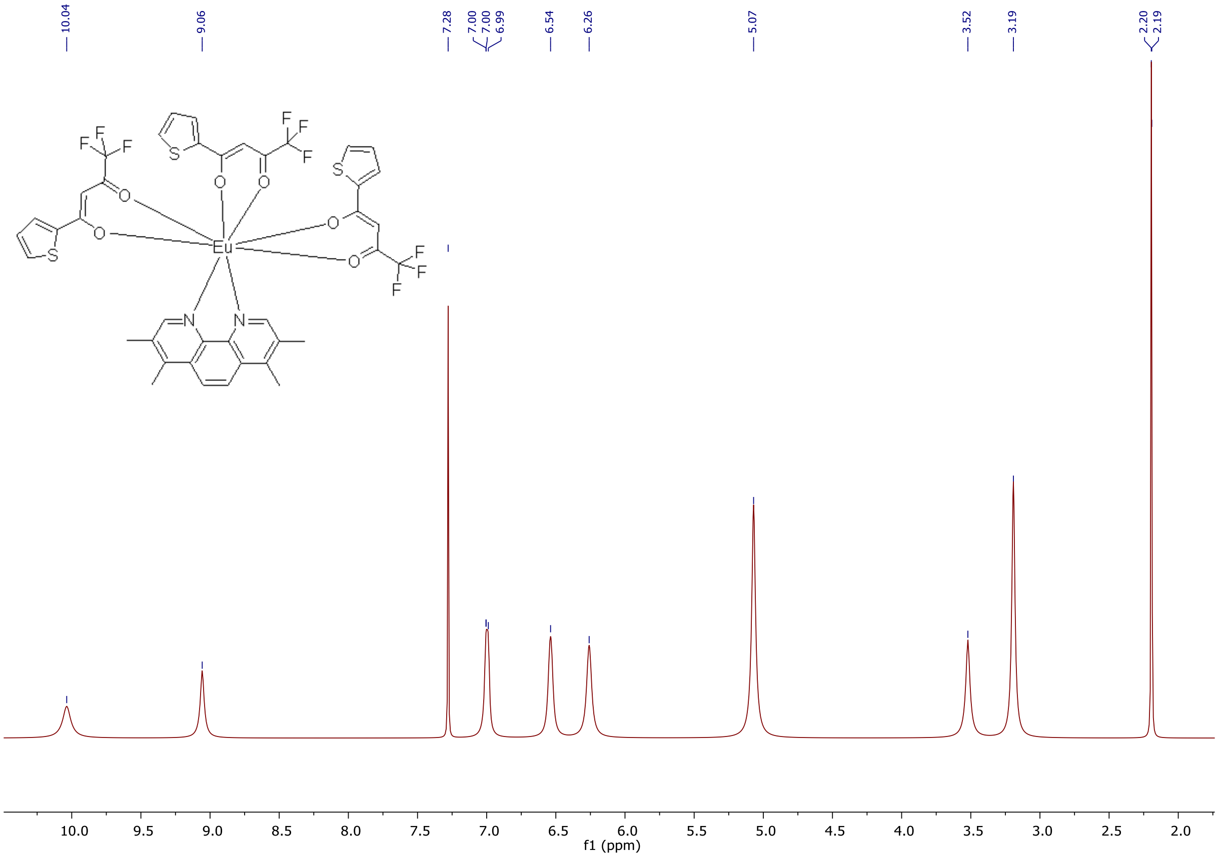

Figure S7: ^{1}H-NMR spectrum of the complex Eu(TTA)$_3$ L$_5$ at 300 MHz in $CDCl_3$.

Eu (TTA)$_3$ L$_5$:^{1}H NMR (300 MHz, $CDCl_3$) δ 10.04 (s, 2H) , 9.06 (s, 2H), 7.28 (s, 3H), 7.00 (dd, J= 2 Hz, 3H), 6.54 (s, 3H), 5.07 (s, 3H), 3.52 (s, 3H), 3.26 (s, 3H), 3.19 (s, 6H), 2.12 (s, 6H).
